# scRNA-seq revealed transcriptional signatures of human umbilical cord primitive stem cells and their germ lineage origin regulated by imprinted genes

**DOI:** 10.1038/s41598-024-79810-4

**Published:** 2024-11-26

**Authors:** Justyna Jarczak, Kamila Bujko, Mariusz Z. Ratajczak, Magdalena Kucia

**Affiliations:** 1 Laboratory of Regenerative Medicine, Center for Preclinical Studies and Technology, Medical University of Warsaw, Ul. Banacha 1B, Warsaw, Poland; 2 Stem Cell Institute at Brown Cancer Center, University of Louisville, 500 S. Floyd Street, Rm. 107, Louisville, KY 40202 USA

**Keywords:** Umbilical cord blood VSELs, Single cell sequencing, RNA sequencing, Adult stem cells, Pluripotency, Transcriptome, Unsupervised clustering, Cell biology, Developmental biology, Immunology, Molecular biology, Stem cells

## Abstract

A population of CD133^+^lin^-^CD45^-^ and CD34^+^lin^-^CD45^-^ very small embryonic-like stem cells (VSELs) has been identified in postnatal human tissues, including bone marrow (BM), mobilized peripheral blood (mPB) and umbilical cord blood (UCB). Under appropriate conditions, VSELs in vitro and in vivo differentiate into tissue-committed stem cells for all three germ layers. Molecular analysis of adult murine BM-purified VSELs revealed that these rare cells deposited during development in adult tissues *(i)* express a similar transcriptome as embryonic stem cells, *(ii)* share several markers characteristic for epiblast and migratory primordial germ cells (PGCs), *(iii)* highly express a polycomb group protein enhancer of zeste drosophila homolog 2 (Ezh2) and finally *(iv)* display a unique pattern of imprinting at crucial paternally inherited genes that promotes their quiescence. Here, by employing single-cell RNA sequencing we demonstrate for the first time that purified from UCB human VSELs defined by expression of CD34 or CD133 antigens and lack of lineage markers, including CD45 antigen express similar molecular signature as murine BM-derived VSELs. Specifically, unsupervised clustering revealed numerous subpopulations of VSELs including ones *i)* annotated to germline compartments, *ii)* regulated by parental imprinting, iii) responding to early developmental fate decisions, *iv)* transcription factors involved in differentiation and development, including homeobox family of genes, and *v)* expressing innate immunity and purinergic signaling genes.

## Introduction

Regenerative medicine is looking for a pluripotent/multipotent stem cells able to differentiate across germ layers. More than 20 years ago our group identified a population of small, early-development stem cells in adult tissues that express pluripotency markers and based on their primitive morphology and gene expression profile, were named very small embryonic-like stem cells (VSELs)^1,2^. The existence of these cells and their differentiation capacity across germ layers was subsequently confirmed by at least 50 other independent groups^3–21^. VSELs and Hematopoietic Stem Cells (HSCS), are detectable in adult bone marrow (BM) and umbilical cord blood (UCB) at very low numbers (~ 10^5^–10^6^). Both stem cell populations can be prospectively isolated to high purity by using antibody cocktail and multiparameter cell sorting, thereby ensuring their subsequent analysis at the single-cell level^22,23^. We and others proposed that VSELs are deposited during embryonic development in a variety of postnatal tissues and organs, including bone marrow (BM), where they reside in a quiescent state^24–26^. They could be mobilized into PB in response to intense exercise, infection, or tissue/organ damage^27,28^. We also reported that their number increases in UCB during delivery^29,30^.

VSELs remain quiescent in a latent state in adult tissues due to epigenetic modifications in the genes involved in insulin/somatotrophic signaling (IGF-IR, IGF-2, Rasgrf1)^24^. Using genome-wide expression analysis and gene set enrichment analysis (GSEA) of a single-cell global transcriptome database, we demonstrated that murine VSELs express at a high-level genes involved in cell cycle checkpoints and genes encoding Polycomb group protein called enhancer of zeste drosophila homolog 2 (Ezh2)^26^. In contrast, they express at low-level genes involved in mitogenic pathways and protein turnover. Moreover, due to strong Ezh2 expression, VSELs have bivalently modified nucleosomes (trimethylated H3K27 and H3K4) at bivalent domains (BDs) in the genes encoding crucial developmental transcription factors (TFs) of the homeobox family^26^. These modifications delay the premature activation of factors regulating the specification of stem cells into several lineages^31^. Notably, BDs modifications are removed during VSELs development by spontaneous or RNA interference-enforced downregulation of Ezh2.

Several independent investigators confirmed our initial data that VSELs can differentiate across germ layers and become specified into tissue-committed stem cell populations. To support this notion, VSELs may be specified for example, in vivo into mesenchymal stem cells^4,32^, cardiomyocytes^33^, endothelial cells^9,34^, pneumocytes type II^13,35^, gametes^36–38^, and, long-term engrafting hematopoietic stem cells (HSCSs)^11,37,39–41^. Nevertheless, more work was needed to examine at the molecular level how primitive VSELs regulate their embryonic-like state and activate lineage specification potential.

In recent years, the development of single-cell RNA-sequencing (scRNA-seq) techniques enabled detailed analysis of transcriptome at the single-cell levels and single-base resolutions. It is feasible to investigate cell population heterogeneity, study the transcriptome of rare cell populations, and identify differential gene expression profiles. To support this single-cell RNA sequencing (scRNA-seq) was employed to identify external signals that regulate transcriptional states in HSCs and, to uncover their heterogeneity state^42–45^. Similarly, it was possible to compare mesenchymal stem cells (MSCs) residing in various tissues, including brain, UCB, adipose tissue, and synovium, as well as to characterize their subpopulations that demonstrate multipotent differentiation into adipogenic, chondrogenic, and osteogenic lineage^46^. Nevertheless, scRNA-seq analysis of transcriptional-based molecular clusters of VSELs to examine their pluripotent character and differentiation potential across three germ layers has not been reported yet.

## Materials and methods

### Umbilical cord blood

Human umbilical cord blood (hUCB) was obtained from a healthy newborn delivered at the Department of Obstetrics and Gynecology, Medical University of Warsaw. The information about sex and ethnicity was not available. This study was performed based on guidelines and approval of the Medical University of Warsaw Bioethics Committee (permission number KB/3/2018). All procedures were performed in accordance with the Declaration of Helsinki (ethical principles for medical research involving human subjects). Informed consent was obtained from all subjects and/or their legal guardian(s).

### Isolation of stem cells

Samples were constantly handled on ice or at 4 °C avoiding cell disruption. hUCB unit, containing a minimum 20 ml was diluted with PBS and carefully layered over Ficoll-Paque (GE Healthcare, Chicago, IL, United States) and centrifuged for 30 min at 400 × g at 4 °C^29^. The mononuclear phase was collected, washed, and used for further analysis.

### Fluorescence activated cell sorting

MNCs were isolated from hUCB unit as described above and subsequently stained with the following antibodies: Lineage (Lin) cocktail of antibodies, each FITC-conjugated: CD235a (clone GA-R2 [HIR2]), anti-CD2 (clone RPA-2.10), anti-CD3 (clone UCHT1), anti-CD14 (clone M5E2), anti-CD16 (clone 3G8), anti-CD19 (clone HIB19), anti-CD24 (clone ML5), anti-CD56 (clone NCAM16.2) and anti-CD66b (clone G10F5) (all BD Biosciences, San Jose, CA, United States); PE-Cy7-conjugated anti-CD45 (clone HI30, BioLegend, San Diego, CA, United States); APC-conjugated anti-CD133 (clone CD133, MiltenyiBiotec, Gladbach, Germany) and PE-conjugated anti-CD34 (clone 581, BioLegend, San Diego, CA, United States). Antibodies were used in the manufacturer’s recommended concentration. Cells were stained in the dark, placed on ice for 30 min, then washed and resuspended in RPMI-1640 medium containing 2% FBS (Corning Inc, Corning, NY, United States). Cells were sorted according to the following strategy: first, small events (2–15 μm in size) were included in the “lymphocyte-like” gate and then further analyzed for the expression of Lin markers and CD45 and CD133 or CD34 antigens. Populations of CD133^+^ VSELs (CD133^+^lin^−^CD45^−^) and HSCS (CD133^+^lin^−^CD45^+^) CD34^+^ VSELs (CD34^+^lin^−^CD45^−^) and HSCSs (CD34^+^lin^−^CD45^+^) were sorted on the MoFlo Astrios EQ cell sorter (Beckman Coulter, Brea, CA, United States).

### Single cell suspension

Directly after sorting, cell quantity, and viability of cell populations were measured, and a cell suspension with 10,000 target cells was used for gel beads preparation with the use of Chromium X Controller (10X Genomics, USA) and Chromium Next GEM Chip G Single Cell Kit (10X Genomics, USA). The procedure consisted of the following steps: GEM Generation and Barcoding, GEM incubation, and Post-GEM-RT Clean-up, after which GEMs were ready for library preparation.

### Single-cell library preparation

Chromium Next GEM Single Cell 3’ GEM, Library & Gel Bead Kit v3.1, and Single Index Kit T Set A (10X Genomics, USA) were used for library preparation according to manufacturer’s guidelines. The procedure consisted of cDNA amplification; cDNA clean-up, cDNA quantification; fragmentation, adapter ligation, post ligation clean-up, sample index PCR, post PCR double sided size selection, library quantification, and quality control. The quantity of libraries was measured with the use of the KAPA Library Quantification Kit (Roche, Switzerland), while quality was verified with a High-Sensitivity DNA Kit 5000 on a TapeStation 4150 (Agilent Technologies, USA). Prepared libraries met quality control criteria for sequencing (Supplementary Fig. 1 and Supplementary Table 1).

### scRNA-seq

Single cell libraries were pooled and run on Illumina NextSeq 1000/2000 (Illumina, San Diego, CA, USA) in P2 flow cell chemistry (200 cycles) with paired-end sequencing mode (read 1–28 bp, read 2–90 bp, index 1 – 10 cycles, index 2 – 10 cycles), assuming 25,000 reads per cell.

### Processing of scRNA-seq data

Raw sequencing data (BCL files) were demultiplexed and converted to fastq files using the bcl2fastq (version v2.20.0.422) within the *10X Genomics Cell Ranger mkfastq pipeline* (CellRanger version 7.2.0; https://www.10xgenomics.com/). Then, Cell Ranger *count* and *aggregate* functions were used for the quality control and processing fastq data. Cells with less than 200 and more than 2500 transcripts, and those with more than 5% of mitochondria-related transcripts were excluded from the analysis. QC metrics are visualized on Supplementary Fig. 2. Sequencing results were mapped to a human genome GRCh38 (version 2020-A) acquired from the 10× Genomics website (https://www.10xgenomics.com/support/software/cell-ranger/downloads). Gene expression measurements for each cell were normalized by the total number of transcripts in the cell, multiplied by a default scale factor, and the normalized values were logtransformed (“*LogNormalize*” method). The log normalized data were reduced to the first 2000 most highly variable genes. Initially, all samples: VSELs (CD133 + lin-CD45-, CD34 + lin-CD45-) and HSCSs (CD133 + lin-CD45 + , CD34 + lin-CD45 +) were analyzed separately with the use of *Cellranger count pipeline* (CellRanger Software, version 7.2.0) to perform alignment, filtering, barcode counting, and UMI counting. Finally, we performed an integrated analysis of two datasets: CD34 + VSELS and CD133 + VSELS, which were merged and analyzed as “pseudobulk”. Downstream analysis was performed with the use of Seurat (version 5.0.1) ^47,48^ and Loupe Browser (version 7.0.1; https://www.10xgenomics.com/).

### Clustering, differential gene expression, and cell markers

Non-linear dimensional reduction to visualize clusters was performed with the use of two methods: two-dimensional t-SNE within Loupe Browser (version 7.0.1)^49^ and uniform manifold approximation and d projection (UMAP) implemented in Seurat (version 5.0.1)^47,48^. It was preceded by PCA analysis, including linear transformation (data scaling), linear dimensional reduction, and PCA visualization and examination. Based on the cell number, expected variability, PCA results and the result of generated ElbowPlot (Supplementary Fig. 3), we next determined the dimensionality of the dataset for all samples (Supplementary Fig. 3), we chose 15 principal components to include in the analysis.

Cell clusters were recognized based on differentially expressed genes, both positive (up-regulated genes) and negative (down-regulated genes) markers, using an adjusted *p*-value < 0.05 and a log2FC > 1. However, for the purposes of this publication, we focused only on positive ones. These genes were subsequently used for the functional analysis and characterization of the identified samples and clusters. Functional analysis was performed with the use of Reactome Pathway Browser^50^. Furthermore, we selected basic cell markers (CD2, CD3, CD4, CD11b (ITGAM), CD14, CD16 (FCGR3A), CD19, CD34, CD41 (ITGA2B), CD45 (PTPRC), CD66b (CEACAM8), CD68, GYPA, CD133 (PROM1), CD117 (c-KIT) as well as genes described as playing important roles in several cellular processes such as: primordial germ cells origin and migration (e. g. Stella, DPPA2, DPPA4); stem cells differentiation (POU5F1, Nanog, SOX1, GATA4); imprinting (e.g. H19, IGF2); DNA methylation (DNMT1, DNMT3a, DNMT3b, DNMT3L); transcription machinery activation and repression (e. g. ERK, RSK, p53, PTEN); chromatin modeling (e. g. BRM, HDAC1); ectoderm, endoderm and mesoderm specification (PAX6, Myf5, SOX7, SOX17); hemostasis (S100A10, ANXA2, CAV1, PPIH), cell surface interaction at the vascular wall (IGKC, IGLC2, IGLC3, IGHM, CD72), thrombin cascade and heart function (GP9, GP1BB, F13A1, PPBP, PF4), heme metabolism (HBA1, HBA2, HBB, UROD, ALAS2), immune system processes (C3, C5, NLRP3, IL1B, IL18) and so on, to determine their expression in CD34+ lin-CD45- and CD133+ lin-CD45-.

Data analysis was done with the use of R^51^, while visualization was performed with the use of the ggplot2 R package (version 3.4.4)^52^.

## Results

### CD133^+^Lin^-^CD45^-^ VSELs are less common in UCB as compared to a CD34^+^Lin^-^CD45^-^ VSELs

Human VSELs are small, lack lineage markers, and express CD34 or CD133 antigens ^29^. Therefore, to purify UCB VSELs we employed multiparameter staining of UCB mononuclear cells (MNCs) and sorted two populations defined as CD34^+^lin^-^CD45^-^ and CD133^+^lin^-^CD45^-^ cells. Supplementary Fig. 4 displays representative dot plots of our sorting strategy.

Briefly, MNCs were separated from the hUCB unit by gradient centrifugation on Ficoll-Paque. Subsequently, MNCs were stained with a cocktail of antibodies as described in Materials and Methods. Next, small objects 2–15 μm in size were included in the lymphocyte gate (Supplementary Fig. 4A) and further analyzed for the expression of lineage markers, where lineage negative events (Lin-) were gated (Supplementary Fig. 4B), and two dot plots were applied to identify the population of CD34 + lin-CD45- VSELs and CD34 + lin-CD45 + HSCs (Supplementary Fig. 4C), as well as CD133 + lin-CD45- VSELs and CD133 + lin-CD45 + HSC (Supplementary Fig. 4D) respectively.

### Transcriptional states in HSCSs and VSELs and evaluation of subpopulations by unsupervised clustering

To explore the composition and diversity of HSCs and VSELs in human UCB, we performed droplet-based scRNA-seq (10 × Genomics, USA) using cells isolated from Ficoll-Paque processed UCB by employing for purification MoFlo Astrios EQ Cell Sorter. We captured a total of 5862 CD133^+^lin^-^CD45^+^, 6811 CD34^+^lin^-^CD45^+^, 3706 CD133^+^lin^-^CD45^-^ and 1592 CD34^+^lin^-^CD45^-^*,* which passed quality control, with a median of 2066, 1613, 457 and 1794 genes per cell, respectively. The more detailed information describing the estimated number of cells detected in all samples and the mean number of reads per cell after quality control is shown in Supplementary Table 2.

**Fig. 1:**
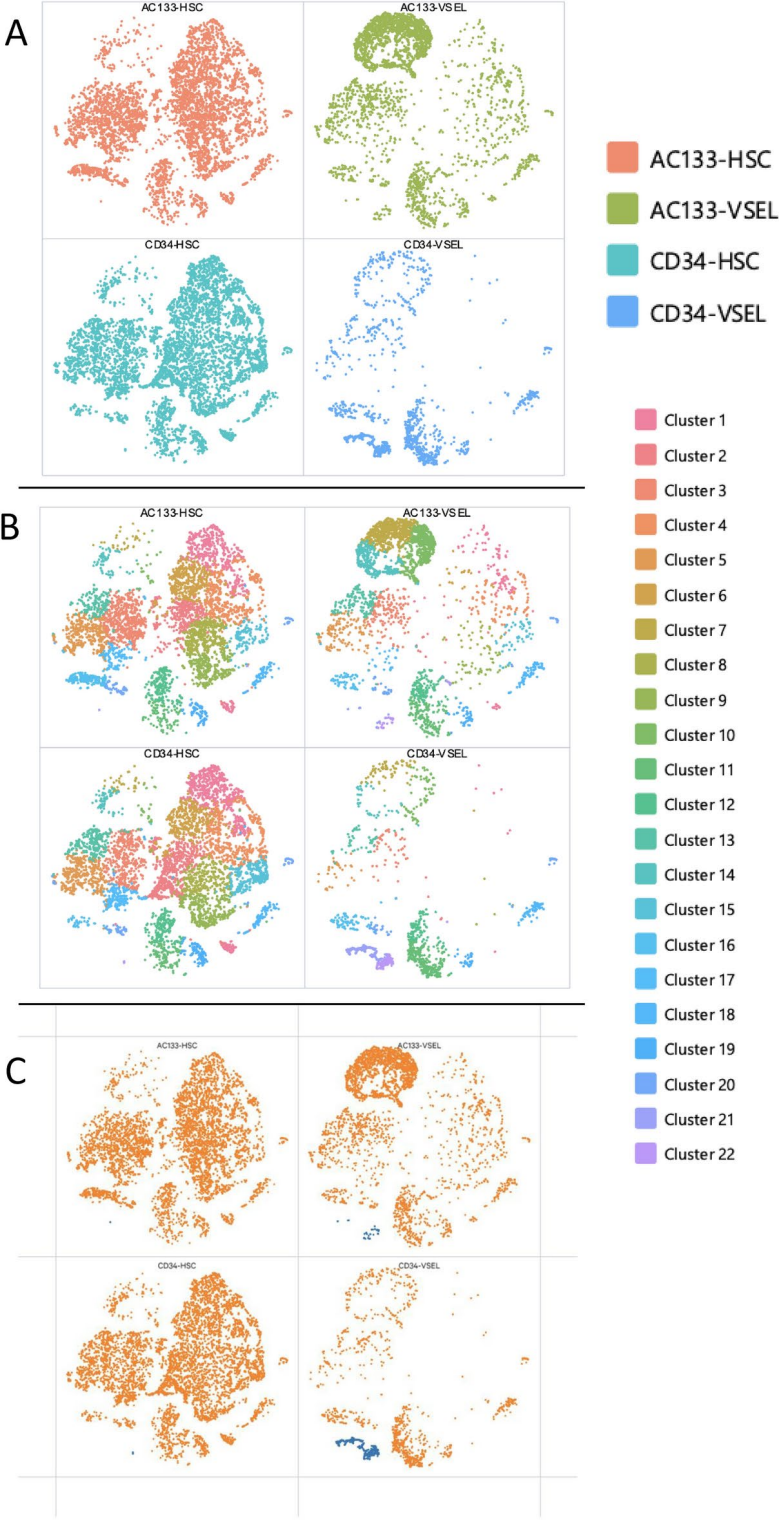
Identification of cell populations (**A**) and subpopulations (clusters) (**B**) in all studied samples: CD34 + lin-CD45-, CD133 + lin-CD45- (VSELs) and CD34 + lin-CD45 + , CD133 + Lin-CD45 + (HSCs) visualized by Loupe Browser 7.0.1 (**A**). Human umbilical cord blood isolated HSCs and VSELs, plot as distinct clusters by a t-SNE method (**B**). Unique subpopulation of both VSEL datasets, more prominent in CD34 + Lin-CD45- (C—marked in blue).

Initially, CD133^+^lin^-^CD45^-^ and CD34^+^lin^-^CD45^-^ VSELs as well as CD133^+^lin^-^CD45^+^ and CD34^+^lin^-^CD45^+^ HSCs were analyzed separately to characterize their diversity and separation into subpopulations by employing CellRanger (CellRanger Software, version 7.2.0) and visualized in Loupe Browser (version 7.0.1). In all the studied samples by the t-SNE method (Loupe Browser 7.0.1), we identified in a bulk cell population (Fig. 1A) 22 subpopulations (graph-based clusters) (Fig. 1B and C). Next, by the uniform manifold approximation and d projection (UMAP) ^53,54^ in the dataset, we identified several cell subpopulations e.g., 13 in CD34^+^lin^-^CD45^+^ (Supplementary Fig. 5A), 11 in CD133^+^lin^-^CD45^+^ (Supplementary Fig. 5B) and 8 in both: CD34^+^lin^-^CD45^-^ (Supplementary Fig. 5C) and CD133^+^lin^-^CD45^-^ VSELs (Supplementary Fig. 5D). Furthermore, additional analysis, where all samples were aggregated into one data set (CellRanger Software, version 7.2.0) and then visualized in Loupe Browser (version 7.0.1) allowed us to confirm the diversity of studied cells but also to identify unique subpopulations exclusive for both VSELs datasets (marked in blue on the Fig. 1C. This subpopulation was more prominent in CD34^+^lin^-^CD45^-^ VSELs (Fig. 1C). We will later characterize at the molecular level this subpopulation while presenting data of differential gene expression between CD133^+^lin^-^CD45^-^ and CD34^+^lin^-^CD45^-^ VSELs datasets.

**Fig. 2:**
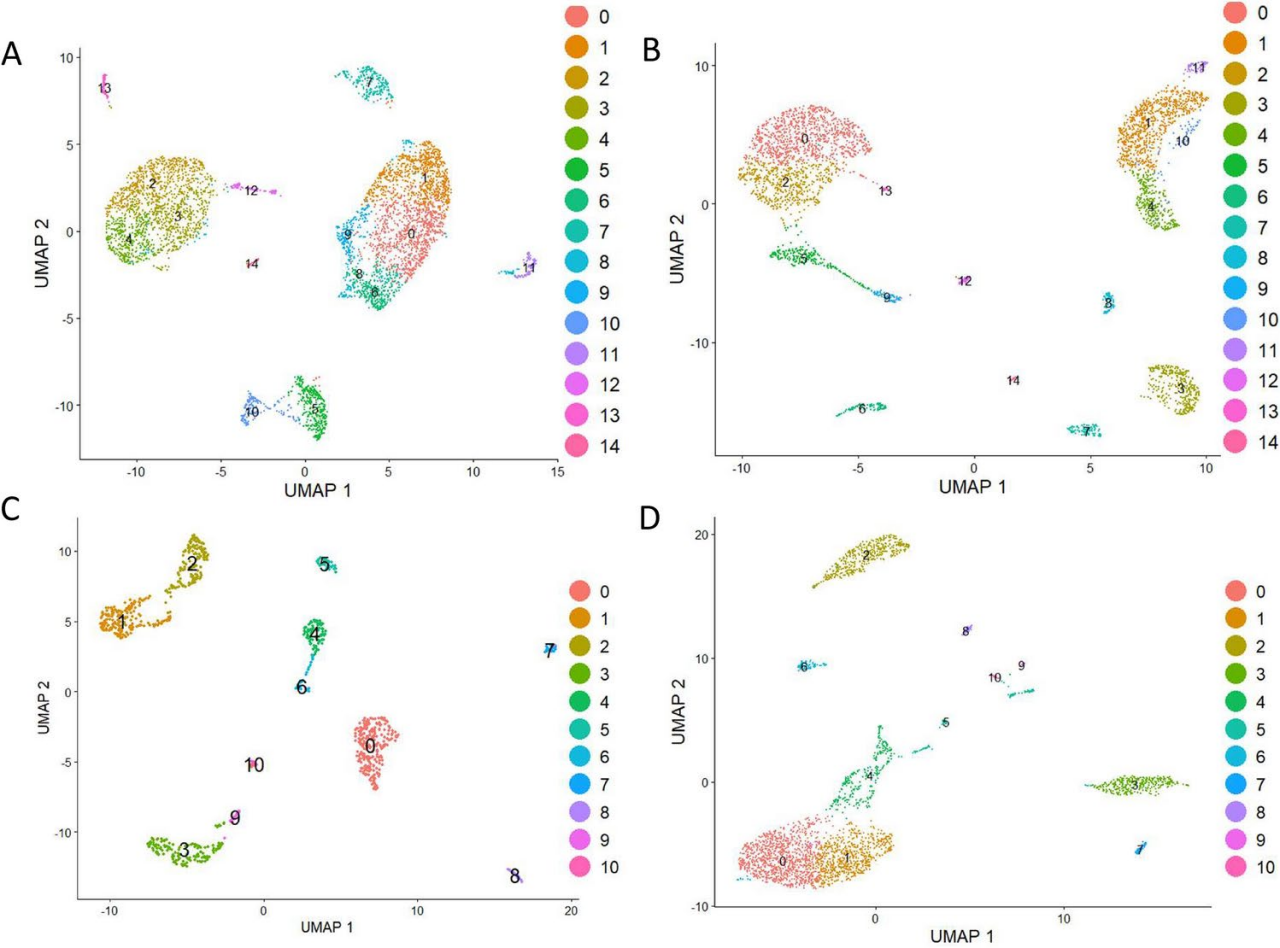
Identification of subpopulations in all studied samples of HSCs (**A** and **B**)—CD34 + lin-CD45 + (A), CD133 + lin-CD45 + (**B**), and VSELs (**C** and **D**) CD34 + lin-CD45- (**C**) and CD133 + lin-CD45- (**D**) visualized by UMAP method. The datasets are first analysed without integration. The resulting clusters are defined both by cell type Uniform manifold approximation and projection (UMAP) plot of HSC and VSELs clusters (n = 1592 cells of CD34 + Lin-CD45- and n = 3706 cells of CD133 + Lin-CD45-).

**Fig. 3:**
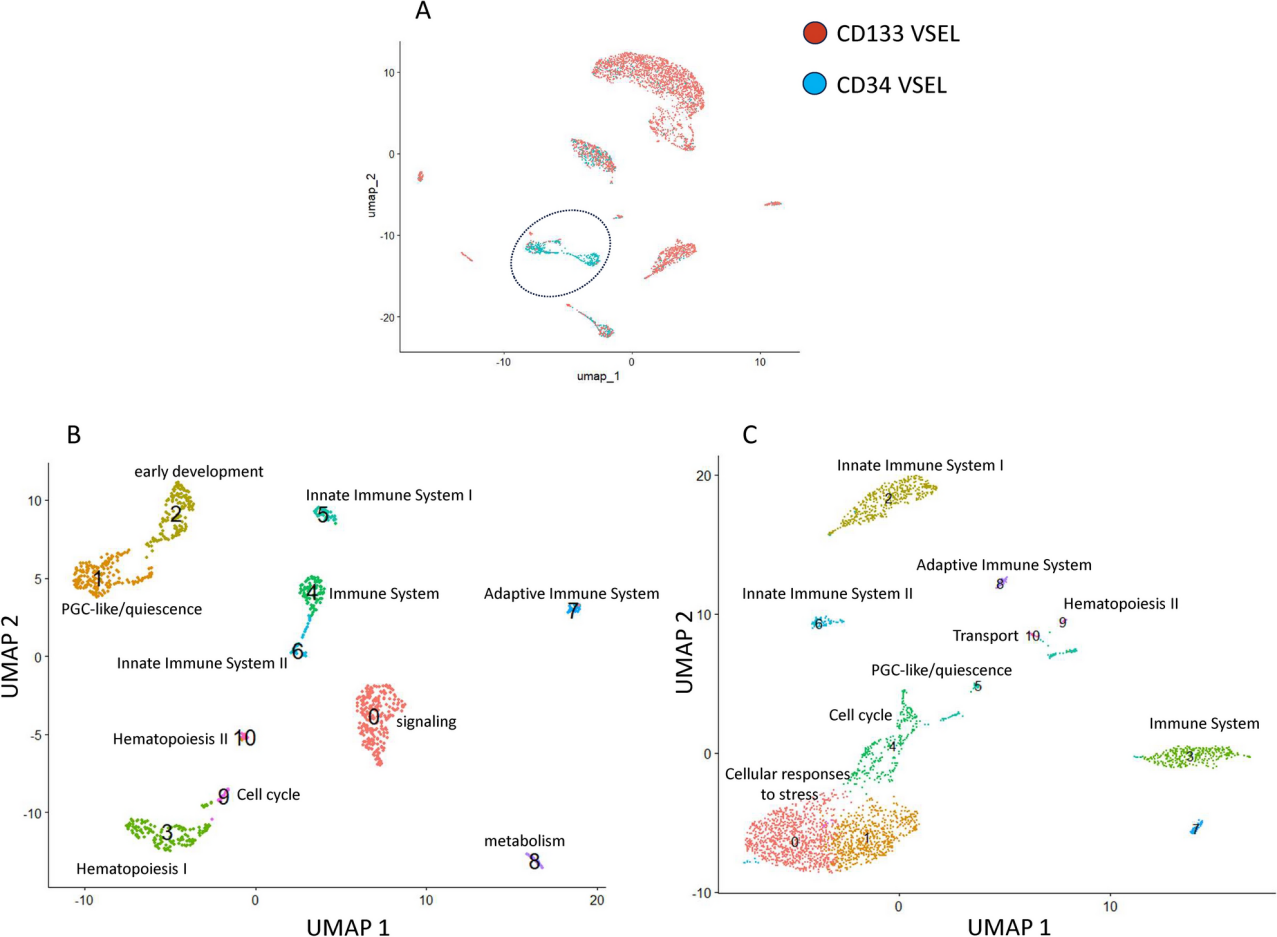
Integration analysis of VSEL libraries defined by CD34 + lin-CD45- and CD133 + lin-CD45- phenotypes (**A**). Integrating two scRNA-seq datasets together (CD34 + lin-CD45- and CD133 + lin-CD45-) idientiefied shared/homologous regions and visualized differences between two subtypes of VSELs with indication of unique clusters 1 and 2 found in CD34 + lin-CD45- (marked by circle on Panel A). Based on Reactome and gene ontology (GO) term pathway enrichment analysis of top 50 up-regulated genes, manual curation of enriched genes was performed to assign labels to each VSEL cluster or state of CD34 + lin-CD45- (**B**) and CD133 + lin-CD45- phenotypes (**C**). Unintegrated UMAP with Leiden clustering of two VSELs scRNA-seq datasets. Note the PGC-like/quiescent clusters on Panel B that corresponds to marked by circle clusters 1 of Fig. 1A.

To obtain more information on differential gene expression in the identified VSELs subpopulations, a detailed analysis was performed employing Seurat (version 5.0.1). This time, the dimensional reduction was based on the UMAP^53,54^. An advantage of UMAP over t-SNE, is that the former clearly separates cluster groups of similar genes from each other, allowing for a better and faster preservation of the data’s structure^53,54^. All samples were analyzed separately, and cell subpopulations were identified and subjected to gene expression analysis to find differentially expressed ones. The number of clusters identified with UMAP projection (Fig. 2) was slightly different in comparison to t-SNE method implemented before in Loupe Browser (version 7.0.1) and comprised of 14 clusters identified for HPCs (CD34^+^lin^-^CD45^+^ and CD133^+^lin^-^CD45^+^*)* (Fig. 2A and B**)** and 10 clusters identified for VSELs (CD34^+^lin^-^CD45^-^ and CD133^+^lin^-^CD45-) (Fig. 2C and D)*.* Based on the results from both methods: t-SNE and UMAP, a higher level of homogeneity was observed in both HSCs samples, while in VSELs, the smaller number of clusters and their greater separation suggests higher heterogeneity. The clear separation into distinct clusters in UMAP (Figs. 3A-C), suggests that VSELs clusters might represent transcriptional states of discrete subtypes of primitive cells. Among subpopulations of CD34^+^lin^-^CD45^-^ and CD133^+^lin^-^CD45^-^, we identified clear transcriptional signature of primordial germ cell specification (Fig. 3B and C), early stages of developmental lineage commitment (Fig. 3B), quiescence (Fig. 3B and C), endothelial specification and activation (Fig. 3B and C), early innate and adaptive immune specification (Fig. 3B and C), and megakaryocyte and platelet development (Fig. 3B and C). Furthermore, we identified a unique subpopulation characteristic for CD34^+^VSELs (Fig. 3A).

### Molecular analysis of UCB purified CD34^+^ and CD133^+^ VSELs subpopulation reveals their germ line and hematopoietic specification potential.

To find differentially expressed features (cluster biomarkers), the *FindMarkers* function in Seurat was used to characterize all the subpopulations within CD34^+^VSELs and CD133^+^VSELs. The list of positive markers in a single cluster is demonstrated in Tables 1 and 2 (in online) for CD34^+^VSELs and CD133^+^VSELs, respectively. We examined the gene markers that define each cluster, separately for CD34^+^ and CD133^+^ VSELs. The Reactome and Gene Ontology (GO) term pathway enrichment analysis, based on the top 50 up-regulated genes and manual curation of enriched genes allowed us to assign labels to each VSELs cluster or state (Fig. 3B and C).

In CD34+ dataset, we have identified in cluster 0 (Table 1 in online) the expression of IL-7R, CAMP4, CD3D, CD3G, TCF7, LTB, BACH2, SKAP1, TRAC, and CD52. Cluster 1 (Table 1 in online) revealed genes involved in cell quiescence, proliferation, and differentiation, including H19, Igf2, S100A16, NNMT, ANXA2, and GNG11. Interestingly, this cluster also comprised DNA-binding protein inhibitors 1 and 3 (ID1 and ID3), that can bind and inhibit transcriptional activity of basic HLH proteins. Clusters 3 and 10 were enriched for genes regulating megakaryocyte and platelet development (Table 1 in online, Fig. 3B).

We reported in the past that BM purified murine VSELs express several markers indicating their close relationship to migrating primordial germ cells (PGCs)^25^ and, that murine VSELs similar to murine PGCs remain quiescent due to the erasure of some genes regulated by paternal imprinting^24^. Furthermore, we demonstrated that murine BM-derived VSELs like human UCB-purified VSELs may differentiate in appropriate experimental models into HSCs^39,40^. Therefore, we focused on changes in PGCs-related imprinted genes in CD34^+^ and CD133^+^ VSELs and their hematopoiesis specification potential.

As expected the prominent cluster 1 for CD34^+^VSELS expressed genes regulated by paternal imprinting being responsible for quiescence of VSELs such as: H19 (avg_log2FC = 2.75; adj. p value = 2.26E-89) and IGF2 (avg_log2FC = 0.95; adj. p value = 2.28E-51) and displayed a decreased expression level of IGF2R (avg_log2FC = -1.57; adj. p value = 6.45E-09) (Table 1 in online). We eliminated the possibility that the “quiescent/germ like” cluster seen in VSELs arose due to contamination of the cell during the sorting procedure of VSELs since HSCs did not display “quiescent/germ like” cluster and the proportions/size of individual subpopulations was different (Fig. 1). Furthermore, the analysis of selected clusters allowed us to find gene patterns differentially expressed between the individual clusters within the same sample.

On the other hand, CD133^+^VSELS cluster 5 (Fig. 3C, Table 2 in online) was characterized by increased expression of imprinted genes: H19 (avg_log2FC = 4.17; adj. p value = 6.68E-177), MEG3 (avg_log2FC = 1.07; adj. *p* value = 1.48E-71), lnc RNA controlled by IGF1 that tightly regulate proliferation SNHG7 (avg_log2FC = 2.06; adj. p value = 2.63E-60), LNCAROD, that is LncRNA activating regulator of DKK1 (avg_log2FC = 0.77; adj. p value = 1.10E-52); neuron fate commitment: ZNF (avg_log2FC = 1.38; adj. *p* value = 3.68E-131), TCF4 (avg_log2FC = 1.83; adj. *p* value = 1.37E-61); epithelial/mesenchymal transition via long non-coding RNA (SNHG8); immunomodulatory: STMN1 (avg_log2FC = 2.76; adj. *p* value = 1.89E-110), RPS3 that is an essential subunit of NF-kappaβ involved in the regulation of key genes in rapid cellular activation responses (avg_log2FC = 2.52; adj. *p* value = 3.53E-52) and, cell division protein kinase 6 (CD6), (Table 2 in online, Fig. 3C).

Thus, scRNA-seq analysis confirms that quiescence/proliferation of both CD34^+^ and CD133^+^ human VSELs is regulated in a similar way, as previously reported for murine cells^24^ by silencing of INS/IGFs signaling. In addition, in both datasets of VSELs we found high expression of RACK1, which is an insulin-like growth factor 1 (IGF-1) receptor-interacting protein that can regulate IGF-1-mediated Akt activation ^55^, and IGF2, an insulin-like growth factor (IGF)-binding protein (IGFBP).

**Fig. 4:**
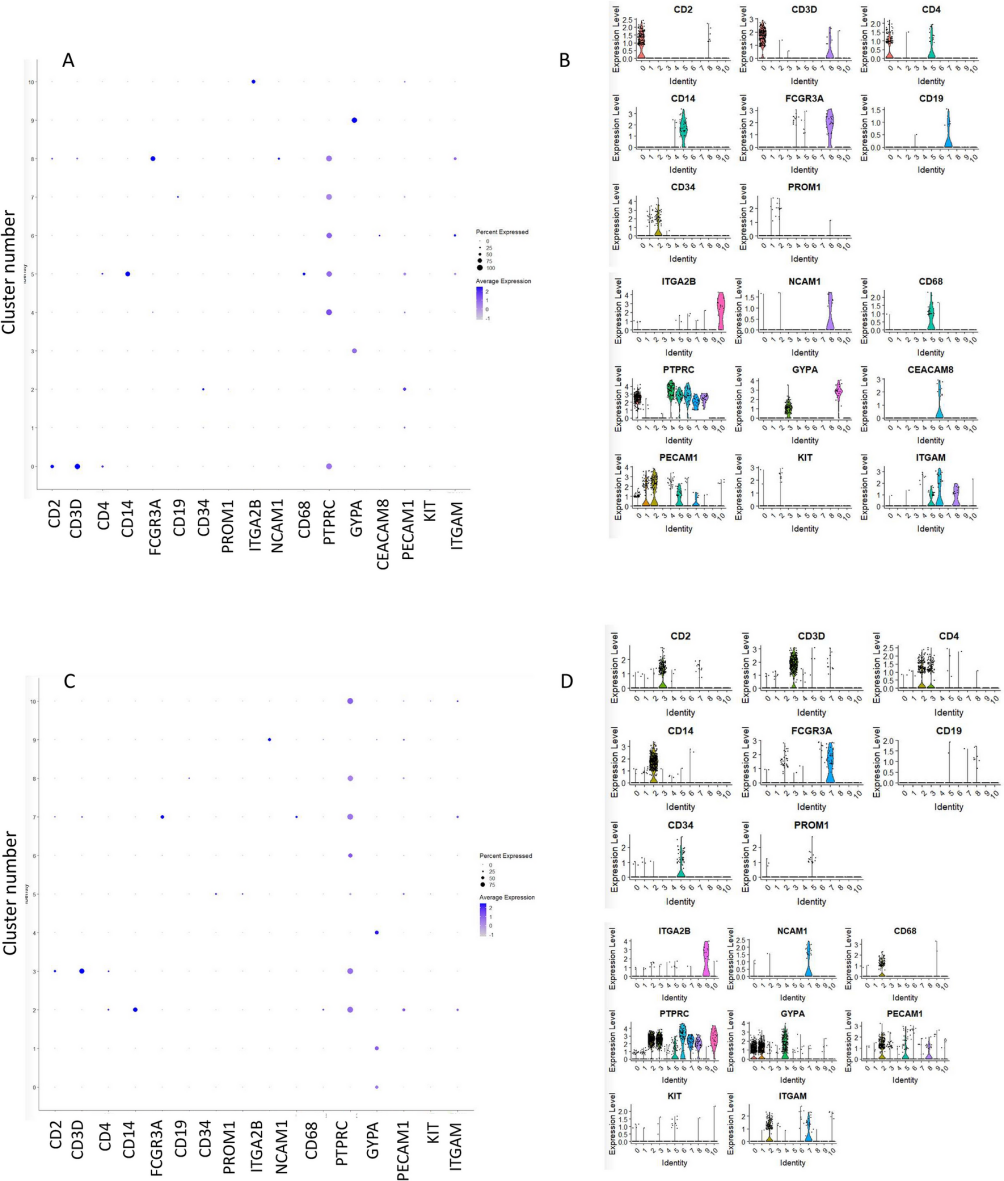
Exploring heterogeneity of VSELs population using Seurat/Expression level of cell markers identified in all clusters of both datasets of VSELs. To identify cell type markers manual analysis of the list of differentially expressed genes in each cluster of CD34 + lin-CD45- and CD133 + lin-CD45- was performed. Expression levels for CD2, CD3D, CD4, CD14, CD16 (FCGR3A), CD19, CD34, CD133 (PROM1), CD41 (ITGA2B), NCAM1, CD68, CD45 (PTPRC), GYPA, CD66b (CEACAM8), PECAM1 (CD45), CD117 (c-KIT) and CD11b (ITGAM), for CD34 + lin-CD45- (Panel A) and CD133 + lin-CD45- (Panel C) visualized by dot plots (Panel A and C) and violin plots in each cluster (Panels B and D).

As mentioned above BM- and UCB-derived VSELs can acquire CD45 antigen expression and in appropriate experimental models could be specified into HSCS^39,40^. To support this, we observed in CD34^+^VSELS in clusters 0, 4, 5, 6, 7, and 8 (Fig. 4A and B), the expression of CD45 (PTPRC). What is important we did not observe the expression of this gene in clusters assigned as “quiescent/germ like” (cluster 1) and “development” (cluster 2) as well as those connected with erythrocytes and platelets functions (clusters 3, 9 and 10). In the remaining clusters of CD34^+^ VSELs, we identified in clusters 0, 5 and 8 the T lymphocyte markers (CD2, CD3, CD4), NK cells markers (CD16 (FCGR3A) and CD56 (NCAM1) in cluster 8; B lymphocyte marker (CD19) in cluster 7; monocyte marker (CD14) in cluster 5; granulocytes (mostly neutrophils) marker (CD66b/CEACAM8) in cluster 6; platelet marker CD41 (ITGA2B) in cluster 10 and, erythrocyte marker (GYPA) in cluster 3 and 9. Furthermore, we identified the expression of mRNA for CD34 in clusters 1 and 2. At the same time, the expression of PROM1 (CD133), which is a very early marker of stem and progenitor cells, was only slightly detectable in single cells of clusters 1 and 2. Additionally, among additional markers for HSCS, we found the expression of PECAM1 (CD31) in clusters 1, 2, 5, and 7 and c-KIT (CD117) in a few cells in cluster 2 (Fig. 4A and B). Notably, clusters 1 and 2 of CD34^+^ dataset (Fig. 4B), which are characterized by transcriptome of early developmental and primordial specification genes, are the only ones that express both CD34 and PROM1 (CD133) (Fig. 4A and B).

**Fig. 5:**
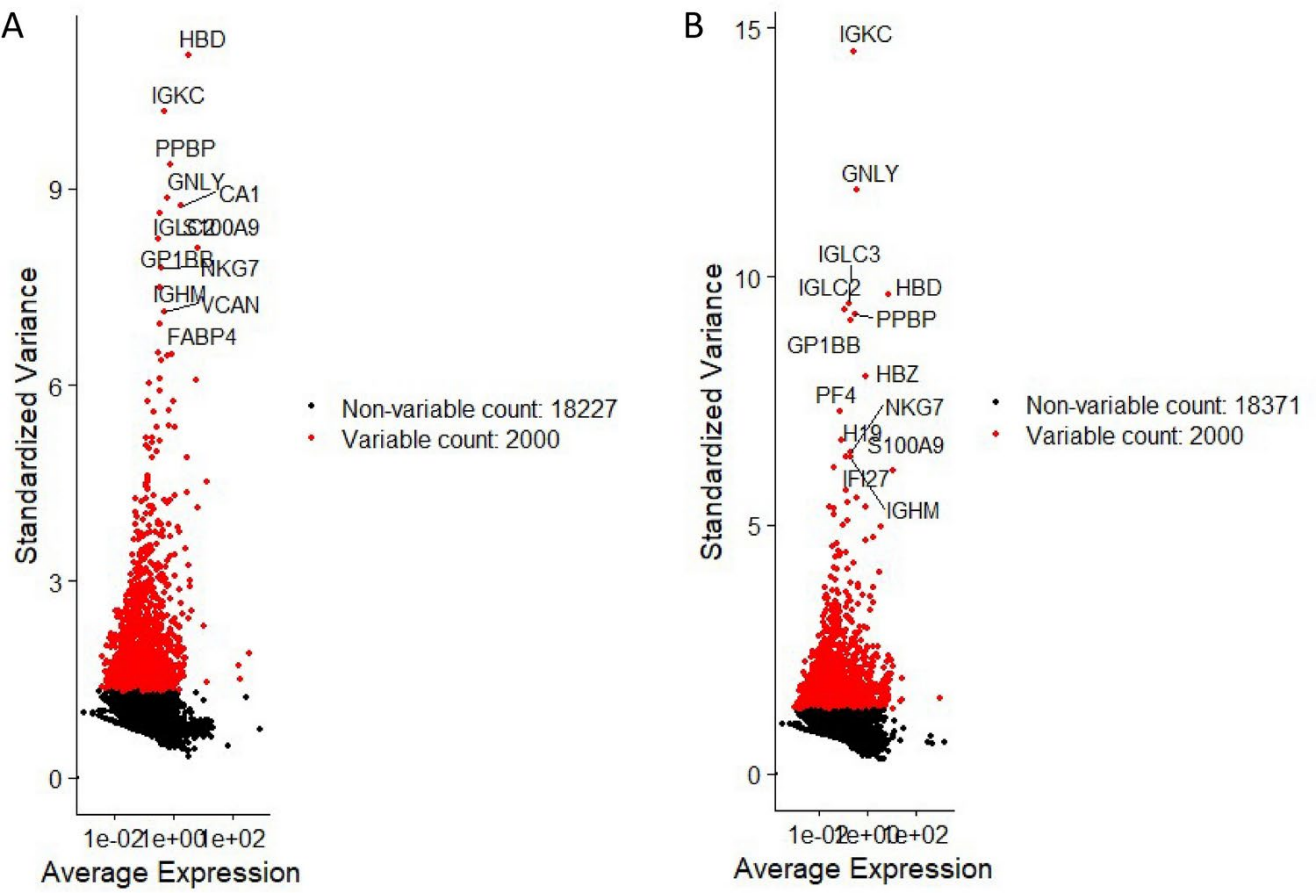
Identification of highly variable genes in VSELs datasets. Volcano plots displaying differences in gene expression among CD34 + lin-CD45- (Panel A) and CD133 + lin-CD45- (Panel B). Genes shown in red represents variable genes and in black – non variable counts.

Similarly, in CD133^+^VSELs data sets (Fig. 4C and D), the expression of several markers was confirmed, however with slight differences as compared to CD34^+^VSELS. The expression of mRNA for CD45 (PTPRC) was found in almost all clusters except for clusters 4 and 9, corresponding to erythrocyte and platelet specification, respectively. Notably, cluster 5 of CD133^+^ VSELs dataset, which constitute of most primitive subset of cells, correlated with the expression of both CD34 and CD133 (Fig. 4C and D). Moreover, in CD133^+^ VSELs we identified clear transcriptional signature of cells expressing fetal hemoglobin F subunit gamma 1 (HBG1), fetal hemoglobin F subunit gamma 2 (HBG2), GYPA, ITGA9 (Fig. 5A and B, Table 1 and 2 in online). We also identified monocyte progenitor markers MPEG1 and CD33, and mast cell mRNA species encoding GATA2, CD63, and HDC (Table 1 and 2 in online) as well as markers for NK (CD96), T-cell (CD3D), and B cell (CD19, CD79B and FCRL1) development.

Therefore, based on cell markers analysis, we further confirmed as expected that CD34^+^ and CD133^+^ UCB-derived VSELs at further stages of development may become specified to the myeloid (erythrocytes, granulocytes, and monocytes) and lymphoid lineage (lymphocytes T, B and NK cells). The most primitive clusters 1 and 2 for the CD34 + lin-CD45- (Fig. 4A and B) and cluster 5 for CD133 + lin-CD45- (Fig. 4C and D) were characterized by the expression of CD34, PROM1, PECAM1, and c-KIT.

### Global gene expression dynamics and differentially expressed genes (DEG) between CD34^+^VSELs and CD133^+^VSELs

Using Seurat (version 4.4.0) we plotted the top 10 highly variable genes for CD34^+^VSELs and CD133^+^VSELs (Fig. 5A and B). The most highly variable genes in CD34^+^VSELs turned out to be HBD, IGKC, PPBP, GNLY, CA1, IGLC2, GP1BB, S100A9, NKG7 and IGHM (Fig. 5A). All indicated genes regulate hematopoiesis and the immune system. Interestingly, the top 10 most variable genes for CD133^+^VSELs included the similar markers: IGKC, GNLY, HBD, PPBP, IGLC2 and GP1BB (Fig. 5B). Additionally, in CD133^+^VSELs we identified IGLC3, HBZ, PF4 and H19, involved in hematopoietic and immune processes, and H19, which as mentioned above is non-coding RNA and a product of paternally imprinted Igf2-H19 locus giving rise to some microRNAs regulating quiescence of VSELS^24^. Furthermore, in both datasets we identified expression of genes involved in hemostasis, gap junctions, membrane dynamics and repair, fibrinolysis, caveolae formation (e.g., ANXA2, CAV1, S100A10, CALM1, GP6).

**Fig. 6:**
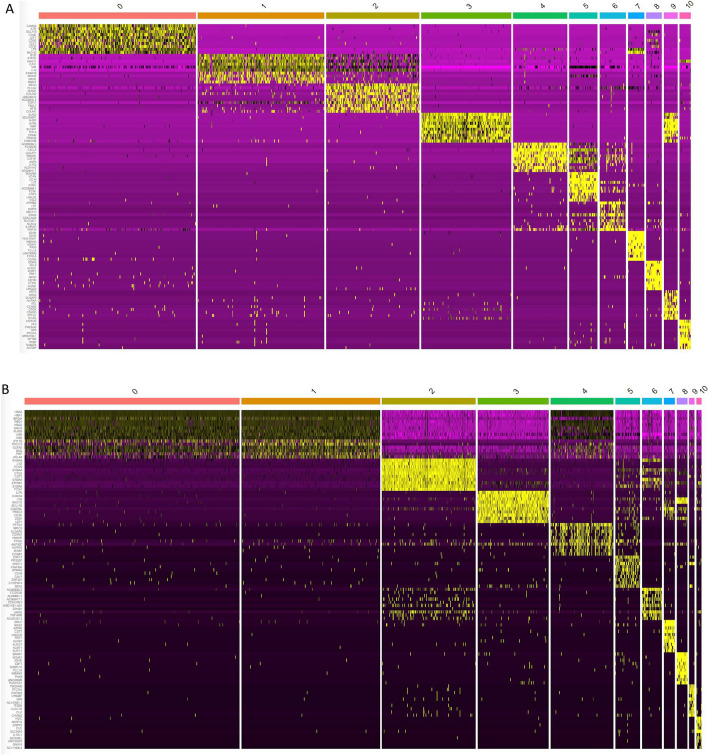
Heatmap of PCA for all clusters showing the expression levels of principal components across different cell clusters identified in the scRNA-seq datasets. The top 10 markers for individual clusters (n = 10) of CD34 + lin-CD45- (Panel A) and CD133 + lin-CD45- (Panel B) were plotted.

**Fig. 7:**
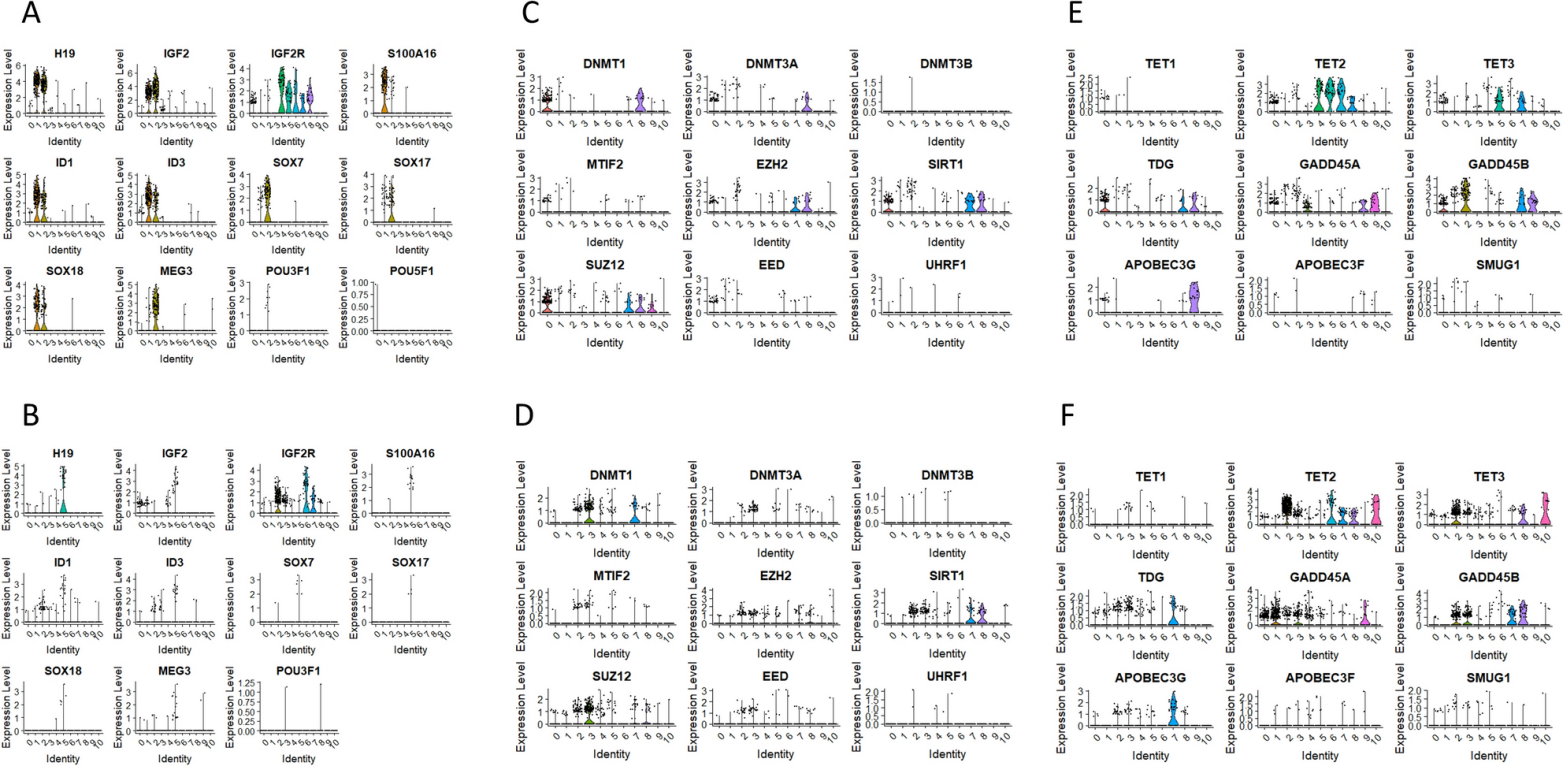
Molecular profile of differentially expressed genes (DEGs) involved in early stages of germ line specification, genomic imprinting, and epigenetic changes both methylation and demethylation processes. Violin plots of H19, IGF2, IGF2R, S100A16, ID1, ID3, Sox7, Sox17, Sox18, Meg2m POU3F1, POUF51, DNMT1, DNMT3a, DNMT3b, MTIF2, Ezh2, SIRT1, SUZ12, EED, UHRF1, TET1, TET2, TET3, TDG, GADD45A, GADD45B, APOBEC3G, APOBEC3F, and SMUG1 expression in CD34 + lin-CD45- (Panels A, C and E) and CD133 + lin-CD45- (Panels B, D and F).

We also generated an expression heatmap for CD34^+^ (Fig. 6A) and CD133^+^ VSELs (Fig. 6B) focusing on clusters. The top 10 markers for each cluster of both data sets confirmed distinct molecular signature except for clusters 1 and 2 for CD34^+^Lin^-^CD45^-^ VSELs (Fig. 6A) and clusters 0 and 1 for CD133^+^Lin^-^CD45^-^ VSELs (Fig. 6B). The observed differences prompted us to conduct a comparison of gene expression between both VSELs populations using the integration analysis in Seurat. The results of the DEG analysis suggest that VSELs comprise several subpopulations with different gene expression levels related to specific functions. Most cells in VSELs clusters 1 and 2 (CD34^+^) and 4 and 5 (CD133^+^) were characterized by a growth repressive profile with high expression of messages related to paternally imprinted H19 and MEG3 genes that are responsible for the quiescent state of VSELs. The growth repressive transcriptome for CD34^+^ VSELs was most pronounced for clusters 1 and 2 including mRNA for H19, Meg3, ID1, and ID3 (Fig. 7A). Also, these clusters were associated with genes involved in the early stages of germ line specification e.g., Sox7, Sox17, and Sox18 (Fig. 7A). For a population of CD133^+^VSELs, a growth repressive transcriptome profile was observed in cluster 5 (Fig. 7B). This finding is consistent with the results of the DEG analysis, in which cells in VSELs clusters 1 and 2 highly express growth repressive genes.

Furthermore, the population of CD34^+^ VSELs specified for proliferation was reflected by the transcriptome profile of methylation/histone modification related genes (Fig. 7C – clusters 7 and 8). Violin plots demonstrated the expression of DNA methyltransferases and sirtuins related to growth restriction/proliferation of cells. For a population of CD133^+^VSELs methylation and histone modification related genes were associated with clusters 3 and 7 (Fig. 7D).

**Fig. 8:**
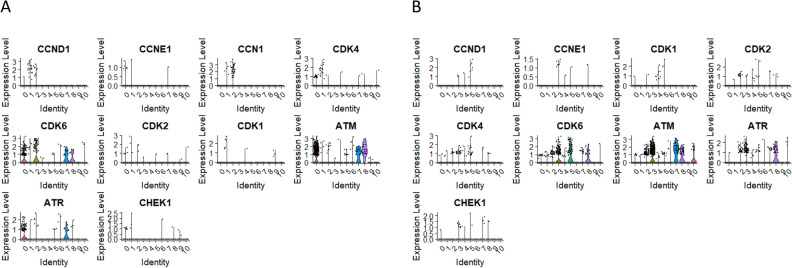
The expression of genes related to cell cycle processes. Violin plots of CCND1, CCNE1, CDK1, CDK2, CDK4, CDK6, ATM, ATR and CHEK1 expression in in CD34 + lin-CD45- (Panel A) and CD133 + lin-CD45- (Panel B).

As mentioned above, we have identified a unique mechanism controlling the biology of murine VSELs in the adult tissues based on the differential expression of imprinted genes regulated by the methylation of the differentially methylated regions (DMRs) in some parentally imprinted genes. In cluster 1 we identified several genes significantly upregulated compared to other subpopulations including, H19, Igf2, Sox7, BMP4, FoxF1, LFNG, EPHA4, SNAI1, GATA-6, MSX1, Sox17, POU3F1, DLL1, TEAD2/4, TCF7L1, MAMLD1, FOXC1, FOXC2, YAP1, and FGF2 (Fig. 7A and C) We also reported that quiescent VSELs to proliferate need to re-establish proper methylation that is erased on paternally imprinted DMRs. This process is regulated by DNMTs and sirtuins^56,57^. As demonstrated by our group, a subset of imprinted and germline developmental genes have their DNA methylation levels closely regulated by Sirt1, which inhibits the Dnmt3L protein at the transcriptional and protein stability levels^58^. Therefore, to shed more light on the quiescence of UCB-derived VSELs we focused in our single-cell cluster analysis on different components of DNMTs and sirtuin signaling. Using scRNA-seq we identified key player(s) involved in the regulation of genome methylation in VSELs clusters, including Ezh2, SIRT1, Suz12, EED, DNMT1, DNMT3A, DNMT3B, and UHRF1 (Fig. 7C and D). Interestingly these genes could be involved in maintaining gene repressive state by polycomb group proteins. Accordingly, some of these proteins form the PRC2 complex containing Ez that catalyzes methylation of histone H3 lysine 27 (H3K37me2/3) and represses its transcription. Importantly, while Ezh1 and Ezh2 maintain repressive chromatin structure^59^, SIRT1 affects DNA methylation of polycomb group protein target genes^60^. Suz12 expression is essential for the activity and integrity of the PRC2 complex and is required for X chromosome inactivation, stem cell maintenance, and differentiation^61^. This is most likely an important additional mechanism regulating VSELs quiescence. Next, we performed an analysis of key genes involved in DNA demethylation, including TET1, TET2, TET3, TDG, GADD45A, GADD45B, APOBEC3G, APOBEC3F, and SMUG1. Interestingly, we detected high expression of TDG, GADD45A, and GADD45B for cluster 1 of CD34+ VSELs with slightly lower expression of TET1, TET2, and TET3 (Fig. 7E). Most primitive cluster 5 of CD133+ VSELs was characterized by the expression of TDG, TET3, and GADD45A (Fig. 7F). Of note GADD45A and GADD45B (Growth Arrest and DNA Damage-Inducible) proteins have been implicated in active DNA demethylation, though their exact mechanism is not entirely understood. Lastly, we analyzed the transcription profile of cyclin-dependent kinases (CDKs), cyclins, and cell cycle checkpoints (ATM, ATR and CHEK1). For cluster 2 in the CD34+ lin-CD45-, we detected no expression of CCNE1, CDK1, CHEK1 and slight expression of CCND1, CCN1, ATM and ATR (Fig. 8A). A similar pattern of transcriptional profile we detected for cluster 5 in CD133+ lin-CD45- (Fig. 8B). Interestingly, we found high expression of CDK6 in cluster 2 of CD34+ lin-CD45-, and cluster 5 of CD133+ lin-CD45-, which is involved in the regulation of quiescence and proliferation of stem cells, stress-induced hematopoiesis, and HSPCs differentiation into myeloid and lymphoid lineages ^62–64^.

**Fig. 9:**
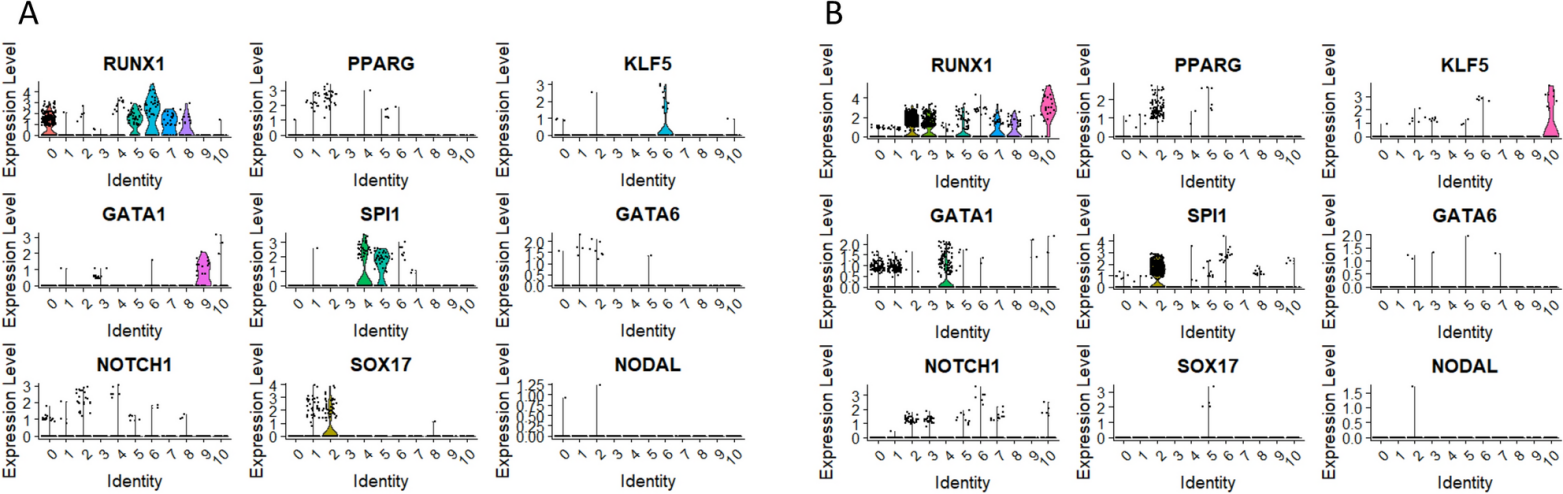
The expression the transcripts associated with ecto, mezo- and endodermal differentiation. Violin plots of RUNX1, PPARG, KLF5, GATA1, SPI1, GATA6, NOTCH1, SOX17 and NODAL expression in CD34 + lin-CD45- (Panel A) and CD133 + lin-CD45- (Panel B).

Finally, we visualized the expression of transcripts associated with ecto, mezo- and endodermal differentiation (RUNX1, PPARG, KLF5, GATA1, SPI1, GATA6, NOTCH1, SOX17 and NODAL) (Fig. 9A and B). In primitive clusters of CD34+ lin-CD45- (cluster 1 and 2), the expression of all genes was observed, however mostly on the slight level. Only, in the case of SOX 17 and PPARG the pronounced expression associated was found (Fig. 9A). Similarly in the case of CD133+ lin-CD45-, the expression of most genes was at quite low levels observed in few cells, only RUNX1 was found to be expressed in primitive cluster (cluster 5) at an increased level (Fig. 9B).

### Integrated analysis of two UCB-purified VSELs populations

**Fig. 10:**
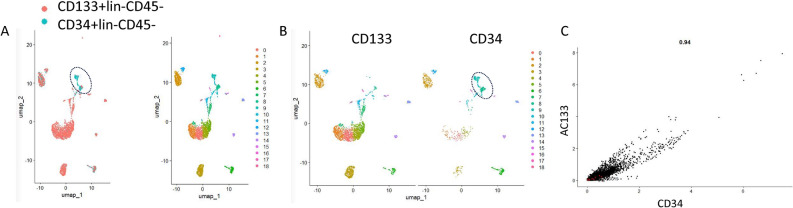
Uniform manifold approximation and projection (UMAP) plots of VSELs clusters. The Seurat v5 integration of two datasets (CD34 and CD133 VSEL) for visualization and unsupervised clustering analysis was performed. Clusters overlay demonstrates similarities in expression profiles of almost all clusters of two datasets, except of cluster 8 and 9 identified for CD34 + lin-CD45- VSELs (Panels A and B), black dotted circle. Scatter plot of linear regression between CD34 and CD133 VSELs datasets showing the relationship between gene expression levels of both VSELs datasets (Panel C).

Finally, we performed an integrated analysis of two datasets of CD34^+^ and CD133^+^ VSELs, which were merged and analyzed as “pseudobulk”. We compared the pseudobulk gene expression profiles and found unique subpopulations characteristic for CD34^+^VSELs (Fig. 10A). Although a strong linear relationship was found (R = 0.94; Fig. 10C), some elements differ between both cell populations at the subpopulation level. Therefore, by employing UMAP we then visualized clusters with the UMAP method and identified unique cells among CD34^+^VSELs as presented in clusters 8 and 9, which are missing in CD133^+^VSELS (Fig. 10B). The list of gene markers for every cluster of CD34^+^VSELs and CD133^+^VSELs is demonstrated in Table 3 in online. These unique clusters among CD34^+^ VSELs were previously shown in dark blue at the Fig. 1C.

**Fig. 11:**
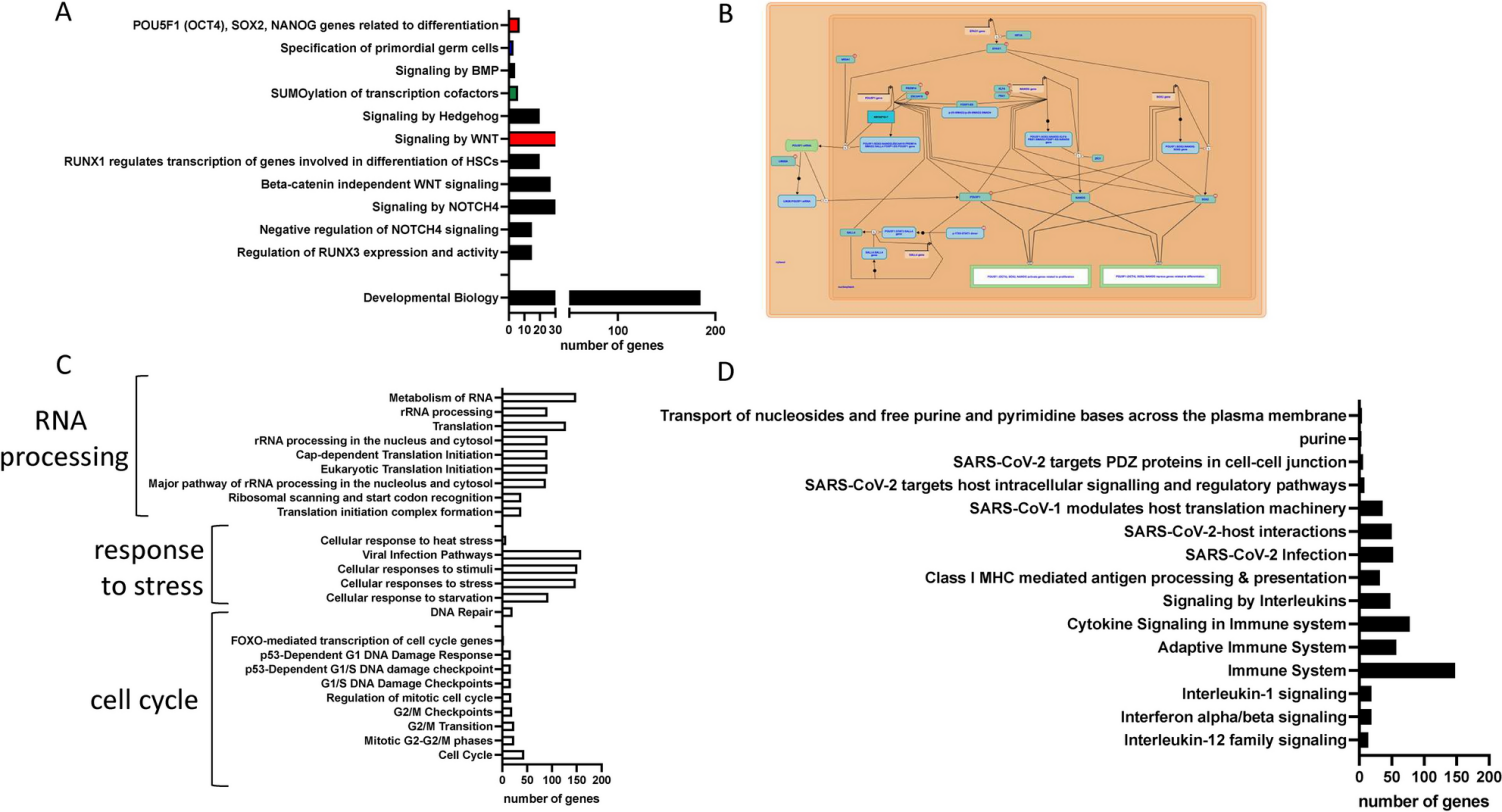
Over-representation of molecular pathways based on DEGs found in cluster 8 in CD34 + lin-CD45- VSELs. Enrichment analysis of transcriptional network of pluripotent stem cells, primordial germ cell specification, SUMO, Wnt, BMP and NOTCH signalling (Panel A and B); RNA processing, responses to external stimuli and cell cycle (Panel C); innate immunity, purinergic signalling and virus-host interaction (Panel D).

To address differences between CD133^+^ and CD34^+^ VSELs we focused on CD34^+^VSELs cluster 8 and identified top 10 genes that are CAV1, NRN1, H19, IFI27, GNG11, IFITM3, NNMT, ID3, BEX3 and IGF2. In the same cluster, we also noticed the expression of FUT9, SPATA18, FOXL1, POU5F1, DLL4, LRFN5, POTEF, PCDH17, GRIK2, and ZNF280A as well as several long-non coding RNAs (lncRNAs) e.g., LINC01058, AL136366.1, LINC02456, AC098934.4 and AL442636.1. Detailed analysis of cluster 8 in CD34^+^ VSELs population revealed also high expression of Sox18, Sox17 characteristics for specification of hemogenic endothelium (HE) (Table 3 in online). Moreover, we detected expression of HOX genes, e.g., HOXA13, HOXB7, HOXA11, HOXB7, and HOXA11, a family of transcription factors that are major regulators of development. We also found expression of CDKN1C (also known as P57kip2) that is a cyclin-dependent kinase inhibitor that functions as a negative regulator of cell proliferation through G1 phase cell cycle arrest. Moreover, we found in CD34^+^ VSELs datasets expression of PRXL2A*,* stress-induced reversible cell-cycle arrest require PRC2/PRC1-mediated control of mitophagy in germline stem cells, and SUMO2*,* involved in safeguarding pluripotency (Fig. 11A). Next, based on the significant expression of genes in CD34^+^VSELs cluster 8, involved in early developmental stages we performed GO analysis and identified genes involved in the transcriptional regulation of stem cells (Fig. 11B), including EPAS1, POU5F1, Nanog, Sox2. Moreover, Reactome analysis revealed the expression of genes involved in PGCs specification (e.g., BMP and STELLA) (Fig. 11A). The results are consistent with those obtained from unintegrated analysis. Of note, our scRNA-seq revealed expression of ZNF706 that as reported is a transcription KLF4 repressor involved in the proliferation of ES cells ^65^.

We also detected high expression of several genes involved in the repression of the cell cycle, response to external stress, and RNA processing (Fig. 11C). CD34^+^ VSELs data sets were characterized by the expression of several genes involved in hematopoietic specification LMO2, S100A6*, SNRPE*, a splicing factor switch controls hematopoietic lineage specification of pluripotent stem cells, and GBP4*,* that regulates transitions in lineage specification and gene regulatory networks in hematopoietic stem/progenitor cells. A subpopulation of CD34^+^ VSELs also express histone variants H3.3 (H3F3B and H3F3A), that maintains adult hematopoietic stem cell homeostasis by enforcing chromatin adaptability^66^ (Table 3 in online).

The high expression of several complement cascade genes (C5, C3, C5aR1, C5aR2, C3aR1), inflammasome (Nlrp3), cytokines (IL1β and IL18) might point to a role of complement in stem cell development^67^ and its role in surveillance to danger associated molecules^68–70^ (Fig. 11D and manuscript in preparation). Moreover, our scRNA-seq analysis of CD34^+^ VSELs data sets detected expression of RPS2, RPS3, RPS3A, RPS5-8, HNRNPA1, EEF1A1, CAV1, UBA52, and SUMO1 which confirms our hypothesis that SARS-Cov19 can infect this primitive population of stem cells (Fig. 11D). The second expression pattern “purinergic signaling” constituted P2X and P2Y related genes that were predominantly found in the “cellular responses to stress” cluster and “innate immune system”. Genes with this signature included CD46, CD55, P2RX4, and P2RX7 (Fig. 11D and manuscript in preparation). This expression of purinergic receptors confirms the role of purinergic signaling in stem cell development^71,72^. Moreover, since VSELs could be specified into mesenchymal stem cells (MSCs) ^4^ and endothelial progenitor cells (EPCs)^9,34^, we detected the expression of several genes involved in MSCs fate via HIF1a and mTOR signaling (DDIT4*)* and EPCs specification (PECAM1; CD31). Moreover, since VSELs may become specified into several types of tissue committed stem cells it is not surprising that we noticed expression of EH domain-containing protein 2 (EHD2), critical gene early stage of adipose-tissue proliferation that is a dynamin-related ATPase influencing several cellular processes, including membrane recycling, caveolae dynamics, and lipid metabolism. We also noticed expression of TBX3 (Table 3 in online) responsible for the specification of neuroepithelial cells. What is also interesting our work revealed the expression of TM4SF18, transmembrane 4 L six family 1 (TM4SF1) member that could be employed for prospective isolation of primitive cells from adult tissues (manuscript in preparation).

Finally, we found CD133^+^ VSELs that revealed the presence of two small clusters – subpopulation of cluster 8 and 10 (Table 3 in online) and we found again H19 (avg_log2FC = 12.44; adj. p value = 9.82E-10), CCN2 (avg_log2FC = 10.49; adj. p value = 9.82E-10), MOX2 (avg_log2FC = 8.84; adj. p value = 0.0035), IGF2 ((avg_log2FC = 8.15; adj. p value = 0.001), NKD2, that is an inhibitor of WNT signaling (avg_log2FC = 7.45; adj. p value = 9.83E-10). Of note, while CCN2 encodes a protein that serves as a mitogen secreted by vascular endothelial cells^73,74^, MEOX2 belongs to mesenchymal Homeobox 2 playing a role in the regulation of myogenesis^75^. Integrated UMAP clusters of CD133 + VSELs datasets visualized also a small subpopulation of cluster 8 with transcriptional signature of developmentally early genes, comprising of DLX6, a member of a homeobox transcription factor gene family playing a role in the forebrain and craniofacial development; paternally-expressed gene 3 PEG3; HOXA11, which encodes a transcription factor involved in proliferation, differentiation, and embryonic development; TCF15 involved in the early transcriptional regulation of mesoderm patterning; H19; IGF1; IGF-2, cyclin dependent kinase inhibitor 1C (CDKN1C); transcription factor involved in neuronal differentiation and/or phenotypic maintenance (ZFP2); NTS that encodes a common precursor for neuromedin N and neurotensin; and NRN1L encoding an extracellular protein that enhances both neurite growth and neuronal survival (Table 3 in online). For that cluster we also detected high expression of SIX4 (avg_log2FC = 9.65; adj. *p* value = 3.96E-15), that is a transcriptional regulator which can act as both a transcriptional repressor and activator by binding a DNA sequence on the target genes and is involved in processes including cell differentiation, migration, and survival^76,77^. Our scRNA-seq data also revealed the expression of sphingosine-1-phosphate receptor 2 (S1PR2) in subpopulation of CD133+ VSELS (Table 3 in online). We also detected similarly as in the case of CD34+ VSELS expression of several genes characteristic for immune system development (manuscript in preparation).

## Discussion

The seminal observation of our single-cell RNA sequencing analysis revealed that CD34^+^ and CD133^+^ VSELs purified from UCB express similar molecular signatures as we reported for murine BM-derived VSELs. Moreover, unsupervised clustering revealed for the first-time numerous subpopulations of UCB-purified VSELs that express genes *i)* annotated to germline compartments, *ii)* regulated by parental imprinting, *iii)* responding to early developmental fate decisions, *iv)* encoding transcription factors involved in differentiation and development, including homeobox family of genes, *v)* expressing innate immunity and purinergic signaling genes and, *iv)* involved the in hematopoietic specification.

We confirmed that human UCB isolated VSELs like what we have shown in the past by genome-wide gene-expression analysis with a small number of highly purified murine bone marrow-derived VSELs^26^ are a heterogenous population of small cells. We and others reported that these quiescent small cells may respond to various stimuli in postnatal organism and become specified across germ layers. Moreover, if they reside in BM, they may be preferentially specified by a specific microenvironment in this organ into hemato/lymphopoietic cells, mesenchymal stem cells and, endothelial progenitors^9,21,32,39,40^. They also express some mRNA species from embryonic development^25^. It is well known that VSELs also circulate a low level in peripheral blood (PB) being mobilized into circulation from hematopoietic and non-hematopoietic tissues. As one can expect, they may also again leave PB and enter BM following BM-derived chemoattractant gradients—that directs migration of HSPCs such as SDF-1, S1P or eATP. Several reports confirmed that VSELs number increases in PB following organ tissue injury, strenuous exercise, and during infection^27,28,78,79^. The number of VSELs is also high in UCB as a result of stress related to delivery^29^. We and others have postulated that VSELs circulating in UCB and neonate PB after delivery may be involved in mending local tissue injuries in neonates after birth^30^. For the same reason we also observed increase of circulating VSELs in maternal PB blood ^30^.

In our pervious report murine BM-purified VSELs expressed (*i)* transcriptome mimicking those observed in murine embryonic stem cells (ESCs), *(ii)* crucial cell cycle checkpoint genes, *(iii)* low level of genes involved in protein turnover and mitogenic pathways, and *(iv)* high level of express enhancer of zeste drosophila homolog 2 (Ezh2)^25,26^. In this current study we observed, a similar pattern of gene expression in both human CD34^+^ and CD133^+^ UCB-purified VSELs what demonstrates developmental homology between these cells isolated from small and large mammals.

We reported for example that murine BM VSELs because of high expression of Ezh2, similarly to ESCs, exhibit bivalently modified nucleosomes (trimethylated H3K27 and H3K4) at promoters of important homeodomain-containing developmental TFs, modulating premature specification of these cells ^26^. In our current experiments analyzing single cell sequencing datasets of UCB-purified VSELs we found expression of similar genes conforming that it would be a universal mechanism across mammals, preventing pre-mature these quiescent cells. Moreover, we observed in the past that the spontaneous downregulation of Ezh2 or its RNA interference-induced downregulation enforced murine VSEL differentiation, as result of removal of the methylation of bivalent at genes regulating tissue specification^26^. In this study we also identified expression of key player(s) involved in regulation of genome methylation in UCB-purified VSELs, including Ezh2, SIRT1, Suz12, EED, DNMT1, DNMT3A, and DNMT3B. These genes are involved in maintaining gene repressive state by polycomb group proteins^60,61,80^. It is known that some of these proteins form the PRC2 complex containing Ez that catalyzes methylation of histone H3 lysine 27 (H3K37me2/3). It explains why Ezh1 and Ezh2 maintain repressive chromatin structure^59,81^. We postulate, a similar mechanism observed for murine VSELs operates most likely in human corresponding VSELs. We conclude that UCB-VSELs like other pluripotent stem cells, might maintain their pluripotent state through an Ezh2-dependent BD-mediated epigenetic mechanism.

We proposed that VSELs originate from cells related to the germline and are deposited in developing organs during embryogenesis, as a backup population for monopotent tissue-committed stem cells^82^. To support this in a current study we found clusters of VSELs that express several genes involved in development of the germ line^25^. Moreover, we also proposed that murine VSELs similarly as PGCs during their migration to genital ridges erase imprinting on some paternally imprinted genes^83–85^ and that this epigenetic mechanism regulates their quiescence^24^. Analyzing our datasets for UCB-VSELs we see a similar pattern in imprinted gene expression as we observed for murine VSELs. Therefore, the erasure of regulatory sequences for development crucial paternally imprinted genes (e.g., at the Igf2–H19 locus) protects these cells from insulin/insulin-like growth factor stimulation could be in addition to the bivalently modified nucleosomes at promoters of important homeodomain-containing developmental TFs, a leading mechanism preventing these cells from the premature depletion from the adult tissues.

Mammalian epigenetic information is largely unchangeable in differentiated somatic cells but completely changed in primordial germ cells (PGCs) and early embryos^83,86–88^. In the current study, we confirm similarities between human VSELs isolated from umbilical cord blood with germ cell compartment, including expression of DNA methyltransferase and demethylases. While the activation of the pluripotency network in PGCs and early embryos is closely tied to epigenetic reprogramming, it was proposed that the expression of the pluripotency network may be associated with the demethylation of some of the Tet1 targets^89^. Recent data has indicated that TDG (Thymine DNA Glycosylase) may be involved in active promoter demethylation. TDG loading on these promoters was synchronized with the demethylation phase, and dynamic cycles of DNA methylation and demethylation have been reported^90,91^. TDG is known to interact with the de novo DNA methyltransferases DNMT3a^92^ and DNMT3b^93^. Notably, TDG inactivation in the murine germ line was associated with embryonic lethality^94^.

The quiescence of UCB-VSELs could be also affected by Hox genes that are a family of transcription factors regulating creation of developmental pattern^95^. These genes are composed of a homeodomain DNA-binding motif, and act in an anti-proliferative manner, with the more potent posterior Hox genes^96^. Our integrated analysis revealed in studied VSELs strong expression of *HOXA13, HOXB7, HOXA11, HOXB7*, and *HOXA11* that further explains their quiescent character in steady state conditions in adult tissues. Integrated analysis also revealed expression of cyclin dependent kinase inhibitor 1C (*CDKN1C*) in VSELs. The encoded protein is a negative regulator of cell proliferation and a potent, tight-binding inhibitor of many G1 cyclin/Cdk complexes^97–99^. Our data also revealed low expression of cyclins in both VSELs datasets that can lead to cell cycle arrest, reduced cell proliferation, or cellular quiescence. Low expression of especially Cyclin D, can promote entry and maintenance of the quiescent state, where cells are metabolically active but not actively dividing^100^. These data *in toto* explain reversible proliferative arrest in which VSELs are not actively dividing, and yet retain the capacity to reenter the cell cycle upon receiving an appropriate stimulus.

The significant alterations in cell functions and proliferative status likely result from signal transduction that activates or suppresses proper signaling cascades. The analysis of receptor expression can shed light on the fundamental characteristics of stem cells since certain stimuli are required for stem cells to maintain an undifferentiated state and/or induce differentiation. Significantly, the maintenance of pluripotency is dependent on the SUMO, Notch, Hox, and WNT signaling cascades^101,102^. The expression of mRNA for these pathways may help human VSELs retain their pluripotent-like state.

Recently, we reversed the quiescent state of UCB VSELs in presence of nicotinamde (NMA) that is an inhibitor of SIRT-1^34,103^ and forces cells to expand and proliferate. SIRT-1 inhibits the activity of the DNMT3L, which is required for the re-methylation of the DNMRs to somatic state in paternally imprinted genes^104^. DNMT3L acts together in a complex with DNMT3A or DNMT3B^58^. While analyzing datasets in UCB-derived VSELs we see the expression of DNMT1, DNMT3A, SIRT1, and UHRF1, which is a central player in maintaining DNA methylation during replication and binds to hemimethylated DNA at CpG sites to recruit DNMT1. UHRF1 helps maintain the repression of differentiation-associated genes, ensuring the preservation of pluripotency^105^. Moreover, we detected in our datasets SUMO E3 ligase-protein PIAS1 (Protein inhibitor of activated STAT-1) that inhibits both the transcription of STAT1 and the DNA-methyltransferase DNMT3A^106^. To maintain the quiescent state, VSELs might utilize a similar mechanism of epigenetic modifications involving SUMO, that safeguards pluripotent stem cells^107^.

In addition, we detected several genes involved in tissue specification, e.g., RUNX1, PPARG, GATA1, PU5 NOTCH1, and SOX17. Non-hematopoietic stem cells are a varied population that are mostly found in bone marrow, mobilized peripheral blood, umbilical cord blood and other stromal tissues, according to recent studies^46,108–112^. It has been discovered that a rare subset of these cells has pluripotent traits. Epigenetic profiling to compare the epigenetic landscapes (DNA methylation, histone modifications) of different stem cell populations to determine their pluripotency status or lineage commitment would be detrimental in future studies. Also, to directly evaluate the relationship between UCB CD34 + and CD133 + VSELs we are planning to employ cell trajectory analysis to reconstruct the dynamic processes that cells undergo during differentiation, development, or response to stimuli. The goal will be to map how cells transition from one state to another, revealing the progression of gene expression changes as they move through different developmental stages or cellular states.

Next, we also identified in UCB-VSELs genes related to innate immunity and purinergic signaling. This is again evidence that these two-development old regulatory pathways are involved in regulating stem cell biology^113^. The expression of these genes detected herein by scRNA-seq was recently confirmed by us in murine and human purified VSELs by employing regular PCR study^114^. Our current scRNA-seq study and analysis of gene expression datasets also confirmed our previous in vitro and in vivo studies demonstrating that human VSELs similarly as murine ones could be specified into myeloid (erythrocytes, granulocytes, and monocytes) and lymphoid lineages (lymphocytes T, B and NK cells)^40^. Since UCB cells may also grow mesenchymal and endothelial colonies^4,9,34^ our datasets provided evidence for expression of genes that regulate VSELs specification into other stem cell types including MSCs and EPCs^115^. These colonies could be derived from BM-residing stem cells mobilized during delivery into UCB and on the other hand could be a result of mesenchymal and endothelial specification of VSELs.

A relevant observation is also an expression of genes involved in the entry of SARS-COVID19 into the cells. It is in frame with our postulated hypothesis that this virus may infect these very primitive population of stem cells and impair their regenerative potential as well as potentially survive in these cells in the latent form^116,117^. Although medication and immunization are lessening the acute SARS-CoV2 infection’s negative social effects, between 10 and 40 percent of patients still experience symptoms even after the acute infection has resolved^118^. The immunological alterations are present in post-COVID e.g., monocytes are comparable to those that are also present in a variety of cell types and are primarily seen following infections, known as "innate immune memory" or “trained immunity”^119^ . To explain better late consequences of SARS-COVID19 infection further studies are warranted weather this pathogen may persist in the latent state in a population of VSELs and HSPCs.

Our study was performed on UCB-VSELs. It would be important to repeat this investigation with BM-purified VSELs. Since the number of these cells decreases with the age^120^, we can expect some differences in gene expression – for example to see more pro-proliferative potential of VSELs isolated from adult human BM and/or a presence of some peripheral tissues specified phenotypes as result of their specification in BM in response to the history of tissue/organ injuries. In conclusion we provide important evidence that VSELs have a unique molecular signature, and their data sets supports that they could be successfully employed as source of stem cells in regenerative medicine. This become feasible as we were able successfully to expand these very rare cells in well controlled ex vivo expansion cultures^34,121^. The fundamental more detailed characteristic of UCB- and BM- purified VSELs would be clarified in future studies using simultaneous profiling of DNA accessibility and gene expression dynamics with ATAC-Seq and RNA-seq, and such experiments will be performed in our laboratory.

## Supplementary Information

Supplementary Information 1.

Supplementary Information 2.

Supplementary Information 3.

Supplementary Information 4.

Supplementary Information 5.

Supplementary Information 6.

Supplementary Information 7.

Supplementary Information 8.

Supplementary Information 9.

Supplementary Information 10.

## Data Availability

The data sets supporting the results of this article are available in the Sequence Read Archive (SRA) (https://www.ncbi.nlm.nih.gov/sra) repository, and assigned unique persistent identifiers: PRJNA1126429 (https://www.ncbi.nlm.nih.gov/sra/PRJNA1126429) and PRJNA1128409 (https://www.ncbi.nlm.nih.gov/sra/PRJNA1128409).

## Abbreviations

BDs: Bivalent domains
BM: Bone marrow
DEG: Differentially expressed genes
DMRs: Differentially methylated regions
EPCs: Endothelial progenitor cells
GO: Gene ontologies
GSEA: Gene set enrichment analysis
H3K27: Lysine 27 on histone H3 (meaning in text: Trimethylation of lysine 27 on histone H3)
H3K4: Lysine 4 on histone H3 (meaning in text: trimethylation of lysine 4 on histone H3)
HSCs: Hematopoietic stem cells
hUCB: Human umbilical cord blood
lin: Lineage
mPB: Mobilized peripheral blood
MNCs: Mononuclear cells
PCA: Principle component analysis
PGCs: Primordial germ cells
scRNA-seq: Single cell RNA sequencing
TFs: Transcription factors
t-SNE: T-distributed Stochastic Neighbor Embedding
UCB: Umbilical cord blood
UMAP: Uniform manifold approximation and projection
UMI: Unique molecular identifier
VSELs: Very small embryonic-like stem cells

## References

[1] Kucia, M. et al. A population of very small embryonic-like (VSEL) CXCR4(+)SSEA-1(+)Oct-4+ stem cells identified in adult bone marrow. *Leukemia* **20**(5), 857–869 (2006). 16498386 10.1038/sj.leu.2404171

[2] Kucia, M. et al. Morphological and molecular characterization of novel population of CXCR4+ SSEA-4+ Oct-4+ very small embryonic-like cells purified from human cord blood: preliminary report. *Leukemia* **21**(2), 297–303 (2007). 17136117 10.1038/sj.leu.2404470

[3] Shaikh, A., Nagvenkar, P., Pethe, P., Hinduja, I. & Bhartiya, D. Molecular and phenotypic characterization of CD133 and SSEA4 enriched very small embryonic-like stem cells in human cord blood. *Leukemia* **29**(9), 1909–1917 (2015). 25882698 10.1038/leu.2015.100

[4] Havens, A. M. et al. Human very small embryonic-like cells generate skeletal structures, in vivo. *Stem Cells Dev.* **22**(4), 622–630 (2013). 23020187 10.1089/scd.2012.0327 PMC3564465

[5] Lo Sicco, C. et al. Identification of a new cell population constitutively circulating in healthy conditions and endowed with a homing ability toward injured sites. *Sci. Rep.* **5**, 16574 (2015). 26560420 10.1038/srep16574 PMC4642305

[6] Monti, M. et al. A novel method for isolation of pluripotent stem cells from human umbilical cord blood. *Stem Cells Dev.* **26**(17), 1258–1269 (2017). 28583028 10.1089/scd.2017.0012

[7] Chang, Y. J., Tien, K. E., Wen, C. H., Hsieh, T. B. & Hwang, S. M. Recovery of CD45(-)/Lin(-)/SSEA-4(+) very small embryonic-like stem cells by cord blood bank standard operating procedures. *Cytotherapy* **16**(4), 560–565 (2014). 24364909 10.1016/j.jcyt.2013.10.009

[8] Gounari, E. et al. Isolation of a novel embryonic stem cell cord blood-derived population with in vitro hematopoietic capacity in the presence of Wharton’s jelly-derived mesenchymal stromal cells. *Cytotherapy* **21**(2), 246–259 (2019). 30522805 10.1016/j.jcyt.2018.11.006

[9] Guerin, C. L. et al. Bone-marrow-derived very small embryonic-like stem cells in patients with critical leg ischaemia: evidence of vasculogenic potential. *Thromb. Haemost.* **113**(5), 1084–1094 (2015). 25608764 10.1160/TH14-09-0748

[10] Howell, J. C. et al. Pluripotent stem cells identified in multiple murine tissues. *Ann. N Y Acad. Sci.* **996**, 158–173 (2003). 12799294 10.1111/j.1749-6632.2003.tb03244.x

[11] Hwang, S. et al. Nonmarrow hematopoiesis occurs in a hyaluronic-acid-rich node and duct system in mice. *Stem Cells Dev.* **23**(21), 2661–2671 (2014). 24914588 10.1089/scd.2014.0075 PMC4201297

[12] Igura, K., Okada, M., Kim, H. W. & Ashraf, M. Identification of small juvenile stem cells in aged bone marrow and their therapeutic potential for repair of the ischemic heart. *Am. J. Physiol. Heart Circ. Physiol.* **305**(9), H1354-1362 (2013). 23997098 10.1152/ajpheart.00379.2013 PMC3840241

[13] Kassmer, S. H. et al. Very small embryonic-like stem cells from the murine bone marrow differentiate into epithelial cells of the lung. *Stem Cells* **31**(12), 2759–2766 (2013). 23681901 10.1002/stem.1413 PMC4536826

[14] Kuruca, S. E., Celik, D. D., Ozerkan, D. & Erdemir, G. Characterization and isolation of very small embryonic-like (VSEL) stem cells obtained from various human hematopoietic cell sources. *Stem Cell Rev. Rep.* **15**(5), 730–742 (2019). 31172457 10.1007/s12015-019-09896-1

[15] Paczkowska, E. et al. Clinical evidence that very small embryonic-like stem cells are mobilized into peripheral blood in patients after stroke. *Stroke* **40**(4), 1237–1244 (2009). 19246697 10.1161/STROKEAHA.108.535062

[16] Wu, J. H., Wang, H. J., Tan, Y. Z. & Li, Z. H. Characterization of rat very small embryonic-like stem cells and cardiac repair after cell transplantation for myocardial infarction. *Stem Cells Dev.* **21**(8), 1367–1379 (2012). 22032240 10.1089/scd.2011.0280

[17] Virant-Klun, I., Skerl, P., Novakovic, S., Vrtacnik-Bokal, E. & Smrkolj, S. Similar population of CD133+ and DDX4+ VSEL-like stem cells sorted from human embryonic stem cell, ovarian, and ovarian cancer ascites cell cultures: The real embryonic stem cells?. *Cells* **8**(7), 706 (2019). 31336813 10.3390/cells8070706 PMC6678667

[18] Jadczyk, T. et al. Effects of trans-endocardial delivery of bone marrow-derived CD133+ cells on angina and quality of life in patients with refractory angina: A sub-analysis of the REGENT-VSEL trial. *Cardiol. J.* **25**(4), 521–529 (2018). 30211929 10.5603/CJ.2018.0082

[19] Nakatsuka, R. et al. Identification and characterization of lineage(-)CD45(-)Sca-1(+) VSEL phenotypic cells residing in adult mouse bone tissue. *Stem Cells Dev.* **25**(1), 27–42 (2016). 26595762 10.1089/scd.2015.0168

[20] Sovalat, H. et al. Identification and isolation from either adult human bone marrow or G-CSF-mobilized peripheral blood of CD34(+)/CD133(+)/CXCR4(+)/ Lin(-)CD45(-) cells, featuring morphological, molecular, and phenotypic characteristics of very small embryonic-like (VSEL) stem cells. *Exp. Hematol.* **39**(4), 495–505 (2011). 21238532 10.1016/j.exphem.2011.01.003

[21] Leppik, L. et al. Role of adult tissue-derived pluripotent stem cells in bone regeneration. *Stem Cell Rev. Rep.* **16**(1), 198–211 (2020). 31828580 10.1007/s12015-019-09943-x PMC6987071

[22] Suszynska, M. et al. The proper criteria for identification and sorting of very small embryonic-like stem cells, and some nomenclature issues. *Stem Cells Dev.* **23**(7), 702–713 (2014). 24299281 10.1089/scd.2013.0472 PMC3967357

[23] Shin, D. M., Suszynska, M., Mierzejewska, K., Ratajczak, J. & Ratajczak, M. Z. Very small embryonic-like stem-cell optimization of isolation protocols: an update of molecular signatures and a review of current in vivo applications. *Exp. Mol. Med.* **45**(11), e56 (2013). 24232255 10.1038/emm.2013.117 PMC3849570

[24] Shin, D. M. et al. Novel epigenetic mechanisms that control pluripotency and quiescence of adult bone marrow-derived Oct4(+) very small embryonic-like stem cells. *Leukemia* **23**(11), 2042–20513 (2009). 19641521 10.1038/leu.2009.153 PMC2783188

[25] Shin, D. M. et al. Molecular signature of adult bone marrow-purified very small embryonic-like stem cells supports their developmental epiblast/germ line origin. *Leukemia* **24**(8), 1450–1461 (2010). 20508611 10.1038/leu.2010.121

[26] Shin, D. M. et al. Global gene expression analysis of very small embryonic-like stem cells reveals that the Ezh2-dependent bivalent domain mechanism contributes to their pluripotent state. *Stem Cells Dev.* **21**(10), 1639–1652 (2012). 22023227 10.1089/scd.2011.0389 PMC3376460

[27] Wojakowski, W. et al. Mobilization of bone marrow-derived Oct-4+ SSEA-4+ very small embryonic-like stem cells in patients with acute myocardial infarction. *J. Am. Coll. Cardiol.* **53**(1), 1–9 (2009). 19118716 10.1016/j.jacc.2008.09.029 PMC5536894

[28] Marycz, K. et al. Endurance exercise mobilizes developmentally early stem cells into peripheral blood and increases their number in bone marrow: Implications for tissue regeneration. *Stem Cells Int.* **2016**, 5756901 (2016). 26664409 10.1155/2016/5756901 PMC4655293

[29] Bujko, K., Ciechanowicz, A. K., Kucia, M. & Ratajczak, M. Z. Molecular analysis and comparison of CD34(+) and CD133(+) very small embryonic-like stem cells purified from umbilical cord blood. *Cytometry A* **103**(9), 703–711 (2023). 37246957 10.1002/cyto.a.24767

[30] Sielatycka, K., Poniewierska-Baran, A., Nurek, K., Torbe, A. & Ratajczak, M. Z. Novel view on umbilical cord blood and maternal peripheral blood-an evidence for an increase in the number of circulating stem cells on both sides of the fetal-maternal circulation barrier. *Stem Cell Rev. Rep.* **13**(6), 774–780 (2017). 28849333 10.1007/s12015-017-9763-z PMC5730629

[31] Sachs, M. et al. Bivalent chromatin marks developmental regulatory genes in the mouse embryonic germline in vivo. *Cell Rep* **3**(6), 1777–1784 (2013). 23727241 10.1016/j.celrep.2013.04.032 PMC3700580

[32] Havens, A. M. et al. Human and murine very small embryonic-like cells represent multipotent tissue progenitors, in vitro and in vivo. *Stem Cells Dev.* **23**(7), 689–701 (2014). 24372153 10.1089/scd.2013.0362 PMC3967374

[33] Wojakowski, W. et al. Cardiomyocyte differentiation of bone marrow-derived Oct-4+CXCR4+SSEA-1+ very small embryonic-like stem cells. *Int. J. Oncol.* **37**(2), 237–247 (2010). 20596650 10.3892/ijo_00000671

[34] Domingues, A. et al. Human CD34(+) very small embryonic-like stem cells can give rise to endothelial colony-forming cells with a multistep differentiation strategy using UM171 and nicotinamide acid. *Leukemia* **36**(5), 1440–1443 (2022). 35169243 10.1038/s41375-022-01517-0 PMC9061289

[35] Ciechanowicz, A. K. et al. Bone marrow-derived VSELs engraft as lung epithelial progenitor cells after bleomycin-induced lung injury. *Cells* **10**(7), 1570 (2021). 34206516 10.3390/cells10071570 PMC8303224

[36] Bhartiya, D., Hinduja, I., Patel, H. & Bhilawadikar, R. Making gametes from pluripotent stem cells—A promising role for very small embryonic-like stem cells. *Reprod. Biol. Endocrinol.* **12**, 114 (2014). 25421462 10.1186/1477-7827-12-114 PMC4255929

[37] Shaikh, A., Anand, S., Kapoor, S., Ganguly, R. & Bhartiya, D. Mouse bone marrow VSELs exhibit differentiation into three embryonic germ lineages and germ & hematopoietic cells in culture. *Stem Cell Rev Rep* **13**(2), 202–216 (2017). 28070859 10.1007/s12015-016-9714-0

[38] Virant-Klun, I. Functional testing of primitive oocyte-like cells developed in ovarian surface epithelium cell culture from small VSEL-like stem cells: Can they be fertilized one day?. *Stem Cell Rev Rep* **14**(5), 715–721 (2018). 29876729 10.1007/s12015-018-9832-y

[39] Ratajczak, J. et al. Adult murine bone marrow-derived very small embryonic-like stem cells differentiate into the hematopoietic lineage after coculture over OP9 stromal cells. *Exp. Hematol.* **39**(2), 225–237 (2011). 21034791 10.1016/j.exphem.2010.10.007 PMC3044339

[40] Ratajczak, J. et al. Hematopoietic differentiation of umbilical cord blood-derived very small embryonic/epiblast-like stem cells. *Leukemia* **25**(8), 1278–1285 (2011). 21483440 10.1038/leu.2011.73 PMC3135663

[41] Lahlil, R. et al. VSELs maintain their pluripotency and competence to differentiate after enhanced ex vivo expansion. *Stem Cell Rev. Rep.* **14**(4), 510–524 (2018). 29736843 10.1007/s12015-018-9821-1 PMC6013546

[42] Pellin, D. et al. A comprehensive single cell transcriptional landscape of human hematopoietic progenitors. *Nat. Commun.* **10**(1), 2395 (2019). 31160568 10.1038/s41467-019-10291-0 PMC6546699

[43] Zeng, Y. et al. Tracing the first hematopoietic stem cell generation in human embryo by single-cell RNA sequencing. *Cell Res.* **29**(11), 881–894 (2019). 31501518 10.1038/s41422-019-0228-6 PMC6888893

[44] Konturek-Ciesla, A. et al. Temporal multimodal single-cell profiling of native hematopoiesis illuminates altered differentiation trajectories with age. *Cell Rep.* **42**(4), 112304 (2023). 36961818 10.1016/j.celrep.2023.112304

[45] Kucinski, I. et al. A time- and single-cell-resolved model of murine bone marrow hematopoiesis. *Cell Stem Cell* **31**(2), 244-259e210 (2024). 38183977 10.1016/j.stem.2023.12.001 PMC7615671

[46] Oguma, Y., Kuroda, Y., Wakao, S., Kushida, Y. & Dezawa, M. Single-cell RNA sequencing reveals different signatures of mesenchymal stromal cell pluripotent-like and multipotent populations. *iScience* **25**(11), 105395 (2022). 36339265 10.1016/j.isci.2022.105395 PMC9633745

[47] Satija, R., Farrell, J. A., Gennert, D., Schier, A. F. & Regev, A. Spatial reconstruction of single-cell gene expression data. *Nat. Biotechnol.* **33**(5), 495–502 (2015). 25867923 10.1038/nbt.3192 PMC4430369

[48] Hao, Y. et al. Dictionary learning for integrative, multimodal and scalable single-cell analysis. *Nat. Biotechnol.* **42**(2), 293–304 (2024). 37231261 10.1038/s41587-023-01767-y PMC10928517

[49] van der Maaten, L. & Hinton, G. Visualizing data using t-SNE. *J. Mach. Learn. Res.* **9**, 2579–2605 (2008).

[50] Milacic, M. et al. The reactome pathway knowledgebase 2024. *Nucl. Acids Res.* **52**(D1), D672–D678 (2024). 37941124 10.1093/nar/gkad1025 PMC10767911

[51] Team RC. (2021) R: A language and environment for statistical computing. R Foundation for Statistical Computing.

[52] Wickham, H. (ed.) *ggplot2: elegant graphics for data analysis* (Springer, 2016).

[53] McInnes, L., Healy, J., Saul, N. & Grobberger, L. UMAP: Uniform manifold approximation and projection. *J. Open Sour. Softw.*10.21105/joss.00861 (2018).

[54] Becht, E. et al. Dimensionality reduction for visualizing single-cell data using UMAP. *Nat. Biotechnol.*10.1038/nbt.4314 (2018). 30531897 10.1038/nbt.4314

[55] Kiely, P. A., Sant, A. & O’Connor, R. RACK1 is an insulin-like growth factor 1 (IGF-1) receptor-interacting protein that can regulate IGF-1-mediated Akt activation and protection from cell death. *J. Biol. Chem.* **277**(25), 22581–22589 (2002). 11964397 10.1074/jbc.M201758200

[56] Maatouk, D. M. et al. DNA methylation is a primary mechanism for silencing postmigratory primordial germ cell genes in both germ cell and somatic cell lineages. *Development* **133**(17), 3411–3418 (2006). 16887828 10.1242/dev.02500

[57] Jing, H. & Lin, H. Sirtuins in epigenetic regulation. *Chem Rev.* **115**(6), 2350–2375 (2015). 25804908 10.1021/cr500457h PMC4610301

[58] Heo, J. et al. Sirt1 regulates DNA methylation and differentiation potential of embryonic stem cells by antagonizing Dnmt3l. *Cell Rep* **18**(8), 1930–1945 (2017). 28228259 10.1016/j.celrep.2017.01.074

[59] Margueron, R. et al. Ezh1 and Ezh2 maintain repressive chromatin through different mechanisms. *Mol. Cell* **32**(4), 503–518 (2008). 19026781 10.1016/j.molcel.2008.11.004 PMC3641558

[60] Wakeling, L. A. et al. SIRT1 affects DNA methylation of polycomb group protein target genes, a hotspot of the epigenetic shift observed in ageing. *Hum. Genomics* **9**(1), 14 (2015). 26104761 10.1186/s40246-015-0036-0 PMC4480908

[61] Pasini, D., Bracken, A. P., Jensen, M. R., Lazzerini Denchi, E. & Helin, K. Suz12 is essential for mouse development and for EZH2 histone methyltransferase activity. *EMBO J.* **23**(20), 4061–4071 (2004). 15385962 10.1038/sj.emboj.7600402 PMC524339

[62] Laurenti, E. et al. CDK6 levels regulate quiescence exit in human hematopoietic stem cells. *Cell Stem Cell* **16**(3), 302–313 (2015). 25704240 10.1016/j.stem.2015.01.017 PMC4359055

[63] Scheicher, R. et al. CDK6 as a key regulator of hematopoietic and leukemic stem cell activation. *Blood* **125**(1), 90–101 (2015). 25342715 10.1182/blood-2014-06-584417 PMC4281832

[64] Loeffler, D. CDK6: HSC fate and cell cycle nexus?. *Blood* **144**(2), 126–128 (2024). 38990543 10.1182/blood.2024025085

[65] Ghaleb, A. M. & Yang, V. W. Kruppel-like factor 4 (KLF4): What we currently know. *Gene* **611**, 27–37 (2017). 28237823 10.1016/j.gene.2017.02.025 PMC5391259

[66] Guo, P. et al. Histone variant H3.3 maintains adult haematopoietic stem cell homeostasis by enforcing chromatin adaptability. *Nat. Cell Biol.* **24**(1), 99–111 (2022). 34961794 10.1038/s41556-021-00795-7 PMC9166935

[67] Hawksworth, O. A., Coulthard, L. G., Mantovani, S. & Woodruff, T. M. Complement in stem cells and development. *Semin Immunol.* **37**, 74–84 (2018). 29525104 10.1016/j.smim.2018.02.009

[68] Ratajczak, M. Z. & Kucia, M. Hematopoiesis and innate immunity: an inseparable couple for good and bad times, bound together by an hormetic relationship. *Leukemia* **36**(1), 23–32 (2022). 34853440 10.1038/s41375-021-01482-0 PMC8727304

[69] Ratajczak, M. Z. et al. Intracellular complement (complosome) is expressed in hematopoietic stem/progenitor cells (HSPCs) and regulates cell trafficking, metabolism and proliferation in an intracrine Nlrp3 inflammasome-dependent manner. *Leukemia* **37**(6), 1401–1405 (2023). 37055506 10.1038/s41375-023-01894-0 PMC10244163

[70] Gong, T., Liu, L., Jiang, W. & Zhou, R. DAMP-sensing receptors in sterile inflammation and inflammatory diseases. *Nat. Rev. Immunol.* **20**(2), 95–112 (2020). 31558839 10.1038/s41577-019-0215-7

[71] Adamiak, M. et al. Novel evidence that extracellular nucleotides and purinergic signaling induce innate immunity-mediated mobilization of hematopoietic stem/progenitor cells. *Leukemia* **32**(9), 1920–1931 (2018). 29725032 10.1038/s41375-018-0122-0 PMC6127086

[72] Burnstock, G. The therapeutic potential of purinergic signalling. *Biochem. Pharmacol.* **151**, 157–165 (2018). 28735873 10.1016/j.bcp.2017.07.016

[73] Hall-Glenn, F. et al. CCN2/connective tissue growth factor is essential for pericyte adhesion and endothelial basement membrane formation during angiogenesis. *PLoS One* **7**(2), e30562 (2012). 22363445 10.1371/journal.pone.0030562 PMC3282727

[74] Bradham, D. M., Igarashi, A., Potter, R. L. & Grotendorst, G. R. Connective tissue growth factor: a cysteine-rich mitogen secreted by human vascular endothelial cells is related to the SRC-induced immediate early gene product CEF-10. *J. Cell Biol.* **114**(6), 1285–1294 (1991). 1654338 10.1083/jcb.114.6.1285 PMC2289134

[75] Mankoo, B. S. et al. The concerted action of Meox homeobox genes is required upstream of genetic pathways essential for the formation, patterning and differentiation of somites. *Development* **130**(19), 4655–4664 (2003). 12925591 10.1242/dev.00687

[76] Talukdar, P. D. & Chatterji, U. Transcriptional co-activators: emerging roles in signaling pathways and potential therapeutic targets for diseases. *Signal Transduct. Target. Ther.* **8**(1), 427 (2023). 37953273 10.1038/s41392-023-01651-w PMC10641101

[77] Yajima, H. et al. Six family genes control the proliferation and differentiation of muscle satellite cells. *Exp. Cell Res.* **316**(17), 2932–2944 (2010). 20696153 10.1016/j.yexcr.2010.08.001

[78] Wojakowski, W. et al. Mobilization of CD34/CXCR4+, CD34/CD117+, c-met+ stem cells, and mononuclear cells expressing early cardiac, muscle, and endothelial markers into peripheral blood in patients with acute myocardial infarction. *Circulation* **110**(20), 3213–3220 (2004). 15533859 10.1161/01.CIR.0000147609.39780.02

[79] Kucia, M. et al. Cells enriched in markers of neural tissue-committed stem cells reside in the bone marrow and are mobilized into the peripheral blood following stroke. *Leukemia* **20**(1), 18–28 (2006). 16270036 10.1038/sj.leu.2404011

[80] Singh, S. K. et al. Sirt1 ablation promotes stress-induced loss of epigenetic and genomic hematopoietic stem and progenitor cell maintenance. *J. Exp. Med.* **210**(5), 987–1001 (2013). 23630229 10.1084/jem.20121608 PMC3646499

[81] Laugesen, A., Hojfeldt, J. W. & Helin, K. Molecular mechanisms directing PRC2 recruitment and H3K27 methylation. *Mol. Cell* **74**(1), 8–18 (2019). 30951652 10.1016/j.molcel.2019.03.011 PMC6452890

[82] Ratajczak, M. Z. et al. A novel view of the adult stem cell compartment from the perspective of a quiescent population of very small embryonic-like stem cells. *Circ Res* **120**(1), 166–178 (2017). 28057792 10.1161/CIRCRESAHA.116.309362 PMC5221475

[83] Reik, W. & Walter, J. Genomic imprinting: parental influence on the genome. *Nat. Rev. Genet.* **2**(1), 21–32 (2001). 11253064 10.1038/35047554

[84] Bartolomei, M. S. & Ferguson-Smith, A. C. Mammalian genomic imprinting. *Cold Spring Harb Perspect Biol* **3**(7), a002592 (2011). 21576252 10.1101/cshperspect.a002592 PMC3119911

[85] Delaval, K. & Feil, R. Epigenetic regulation of mammalian genomic imprinting. *Curr Opin. Genet. Dev.* **14**(2), 188–195 (2004). 15196466 10.1016/j.gde.2004.01.005

[86] Durcova-Hills, G. & Surani, A. Reprogramming primordial germ cells (PGC) to embryonic germ (EG) cells. *Curr. Protoc. Stem Cell Biol.*10.1002/9780470151808.sc01a03s5 (2008). 18770625 10.1002/9780470151808.sc01a03s5

[87] Surani, M. A., Hayashi, K. & Hajkova, P. Genetic and epigenetic regulators of pluripotency. *Cell* **128**(4), 747–762 (2007). 17320511 10.1016/j.cell.2007.02.010

[88] Hayashi, K., de Sousa Lopes, S. M. & Surani, M. A. Germ cell specification in mice. *Science* **316**(5823), 394–396 (2007). 17446386 10.1126/science.1137545

[89] Seisenberger, S. et al. The dynamics of genome-wide DNA methylation reprogramming in mouse primordial germ cells. *Mol Cell* **48**(6), 849–862 (2012). 23219530 10.1016/j.molcel.2012.11.001 PMC3533687

[90] Kangaspeska, S. et al. Transient cyclical methylation of promoter DNA. *Nature* **452**(7183), 112–115 (2008). 18322535 10.1038/nature06640

[91] Metivier, R. et al. Cyclical DNA methylation of a transcriptionally active promoter. *Nature* **452**(7183), 45–50 (2008). 18322525 10.1038/nature06544

[92] Li, Y. Q., Zhou, P. Z., Zheng, X. D., Walsh, C. P. & Xu, G. L. Association of Dnmt3a and thymine DNA glycosylase links DNA methylation with base-excision repair. *Nucl. Acids Res* **35**(2), 390–400 (2007). 17175537 10.1093/nar/gkl1052 PMC1802599

[93] Boland, M. J. & Christman, J. K. Characterization of Dnmt3b:thymine-DNA glycosylase interaction and stimulation of thymine glycosylase-mediated repair by DNA methyltransferase(s) and RNA. *J. Mol. Biol.* **379**(3), 492–504 (2008). 18452947 10.1016/j.jmb.2008.02.049 PMC2705441

[94] Dalton, S. R. & Bellacosa, A. DNA demethylation by TDG. *Epigenomics* **4**(4), 459–467 (2012). 22920184 10.2217/epi.12.36 PMC3600859

[95] Deschamps, J. & Duboule, D. Embryonic timing, axial stem cells, chromatin dynamics, and the Hox clock. *Genes Dev.* **31**(14), 1406–1416 (2017). 28860158 10.1101/gad.303123.117 PMC5588924

[96] Zhang, Y., Yu, Y., Su, X. & Lu, Y. HOXD8 inhibits the proliferation and migration of triple-negative breast cancer cells and induces apoptosis in them through regulation of AKT/mTOR pathway. *Reprod. Biol.* **21**(4), 100544 (2021). 34454307 10.1016/j.repbio.2021.100544

[97] Harper, J. W., Adami, G. R., Wei, N., Keyomarsi, K. & Elledge, S. J. The p21 Cdk-interacting protein Cip1 is a potent inhibitor of G1 cyclin-dependent kinases. *Cell* **75**(4), 805–816 (1993). 8242751 10.1016/0092-8674(93)90499-g

[98] Marques-Torrejon, M. A. et al. Cyclin-dependent kinase inhibitor p21 controls adult neural stem cell expansion by regulating Sox2 gene expression. *Cell Stem Cell* **12**(1), 88–100 (2013). 23260487 10.1016/j.stem.2012.12.001 PMC3714747

[99] Cheng, T. & Scadden, D. T. Cell cycle entry of hematopoietic stem and progenitor cells controlled by distinct cyclin-dependent kinase inhibitors. *Int J Hematol* **75**(5), 460–465 (2002). 12095144 10.1007/BF02982107

[100] Forzani, C. et al. WOX5 suppresses CYCLIN D activity to establish quiescence at the center of the root stem cell niche. *Curr. Biol.* **24**(16), 1939–1944 (2014). 25127220 10.1016/j.cub.2014.07.019 PMC4148176

[101] Yu, X. et al. Notch signaling activation in human embryonic stem cells is required for embryonic, but not trophoblastic, lineage commitment. *Cell Stem Cell* **2**(5), 461–471 (2008). 18462696 10.1016/j.stem.2008.03.001 PMC2442567

[102] Sato, N., Meijer, L., Skaltsounis, L., Greengard, P. & Brivanlou, A. H. Maintenance of pluripotency in human and mouse embryonic stem cells through activation of Wnt signaling by a pharmacological GSK-3-specific inhibitor. *Nat. Med.* **10**(1), 55–63 (2004). 14702635 10.1038/nm979

[103] Horwitz, M. E. et al. Umbilical cord blood expansion with nicotinamide provides long-term multilineage engraftment. *J. Clin. Invest.* **124**(7), 3121–3128 (2014). 24911148 10.1172/JCI74556 PMC4071379

[104] Peng, L. et al. SIRT1 deacetylates the DNA methyltransferase 1 (DNMT1) protein and alters its activities. *Mol Cell Biol* **31**(23), 4720–4734 (2011). 21947282 10.1128/MCB.06147-11 PMC3232929

[105] Moore, L. D., Le, T. & Fan, G. DNA methylation and its basic function. *Neuropsychopharmacology* **38**(1), 23–38 (2013). 22781841 10.1038/npp.2012.112 PMC3521964

[106] Liu, Y. et al. Protein inhibitor of activated STAT 1 (PIAS1) is identified as the SUMO E3 ligase of CCAAT/enhancer-binding protein beta (C/EBPbeta) during adipogenesis. *Mol Cell Biol* **33**(22), 4606–4617 (2013). 24061474 10.1128/MCB.00723-13 PMC3838193

[107] Cossec, J. C. et al. SUMO safeguards somatic and pluripotent cell identities by enforcing distinct chromatin states. *Cell Stem Cell* **23**(5), 742-757e748 (2018). 30401455 10.1016/j.stem.2018.10.001

[108] D’Ippolito, G. et al. Marrow-isolated adult multilineage inducible (MIAMI) cells, a unique population of postnatal young and old human cells with extensive expansion and differentiation potential. *J. Cell. Sci.* **117**(14), 2971–2981 (2004). 15173316 10.1242/jcs.01103

[109] Vacanti, M. P., Roy, A., Cortiella, J., Bonassar, L. & Vacanti, C. A. Identification and initial characterization of spore-like cells in adult mammals. *J. Cell Biochem.* **80**(3), 455–460 (2001). 11135375

[110] Jiang, Y. et al. Pluripotency of mesenchymal stem cells derived from adult marrow. *Nature* **418**(6893), 41–49 (2002). 12077603 10.1038/nature00870

[111] Feng, S. W. et al. Small blood stem cells for enhancing early osseointegration formation on dental implants: a human phase I safety study. *Stem Cell Res Ther.* **12**(1), 380 (2021). 34215319 10.1186/s13287-021-02461-z PMC8254299

[112] Aprile, D., Patrone, D., Peluso, G. & Galderisi, U. Multipotent/pluripotent stem cell populations in stromal tissues and peripheral blood: Exploring diversity, potential, and therapeutic applications. *Stem Cell Res Ther.* **15**(1), 139 (2024). 38735988 10.1186/s13287-024-03752-x PMC11089765

[113] Ratajczak, M. Z., Bujko, K., Brzezniakiewicz-Janus, K., Ratajczak, J. & Kucia, M. Hematopoiesis revolves around the primordial evolutional rhythm of purinergic signaling and innate immunity - A journey to the developmental roots. *Stem Cell Rev Rep.* **20**(3), 827–838 (2024). 38363476 10.1007/s12015-024-10692-9 PMC10984895

[114] Bujko, K., Brzezniakiewicz-Janus, K., Jarczak, J., Kucia, M. & Ratajczak, M. Murine and human-purified very small embryonic-like stem cells (VSELs) express purinergic receptors and migrate to extracellular ATP gradient. *Stem Cell Rev Rep.* **20**, 1357–13664 (2024). 38635127 10.1007/s12015-024-10716-4 PMC11222280

[115] Javed, M. J. et al. Endothelial colony forming cells and mesenchymal stem cells are enriched at different gestational ages in human umbilical cord blood. *Pediatr. Res.* **64**(1), 68–73 (2008). 18360305 10.1203/PDR.0b013e31817445e9

[116] Kucia, M. et al. An evidence that SARS-Cov-2/COVID-19 spike protein (SP) damages hematopoietic stem/progenitor cells in the mechanism of pyroptosis in Nlrp3 inflammasome-dependent manner. *Leukemia* **35**(10), 3026–3029 (2021). 34163002 10.1038/s41375-021-01332-z PMC8219510

[117] Ropa, J., Cooper, S., Capitano, M. L., Van’t Hof, W. & Broxmeyer, H. E. Human hematopoietic stem, progenitor, and immune cells respond ex vivo to SARS-CoV-2 spike protein. *Stem Cell Rev. Rep.* **17**, 253–265 (2020). 33089452 10.1007/s12015-020-10056-z PMC7577648

[118] Boes, M. & Falter-Braun, P. Long-COVID-19: the persisting imprint of SARS-CoV-2 infections on the innate immune system. *Signal Transduct Target Ther* **8**(1), 460 (2023). 38097574 10.1038/s41392-023-01717-9 PMC10721820

[119] Cheong, J. G. et al. Epigenetic memory of coronavirus infection in innate immune cells and their progenitors. *Cell* **186**(18), 3882-3902 e3824 (2023). 37597510 10.1016/j.cell.2023.07.019 PMC10638861

[120] Kucia, M. et al. The negative effect of prolonged somatotrophic/insulin signaling on an adult bone marrow-residing population of pluripotent very small embryonic-like stem cells (VSELs). *Age (Dordr)* **35**(2), 315–330 (2013). 22218782 10.1007/s11357-011-9364-8 PMC3592960

[121] Ratajczak, M. Z., Ratajczak, J. & Kucia, M. Very small embryonic-like stem cells (VSELs). *Circ. Res.* **124**(2), 208–210 (2019). 30653438 10.1161/CIRCRESAHA.118.314287 PMC6461217

