# Supplementary material for: scRNA-seq revealed transcriptional signatures of human umbilical cord primitive stem cells and their germ lineage origin regulated by imprinted genes
Source: Sci Rep. 2024 Nov 26;14:29264. doi: 10.1038/s41598-024-79810-4 (PMC11589151; doi:10.1038/s41598-024-79810-4)
Supplement: Supplementary file 1 — Supplementary Information 1. [file 41598_2024_79810_MOESM1_ESM.pdf]

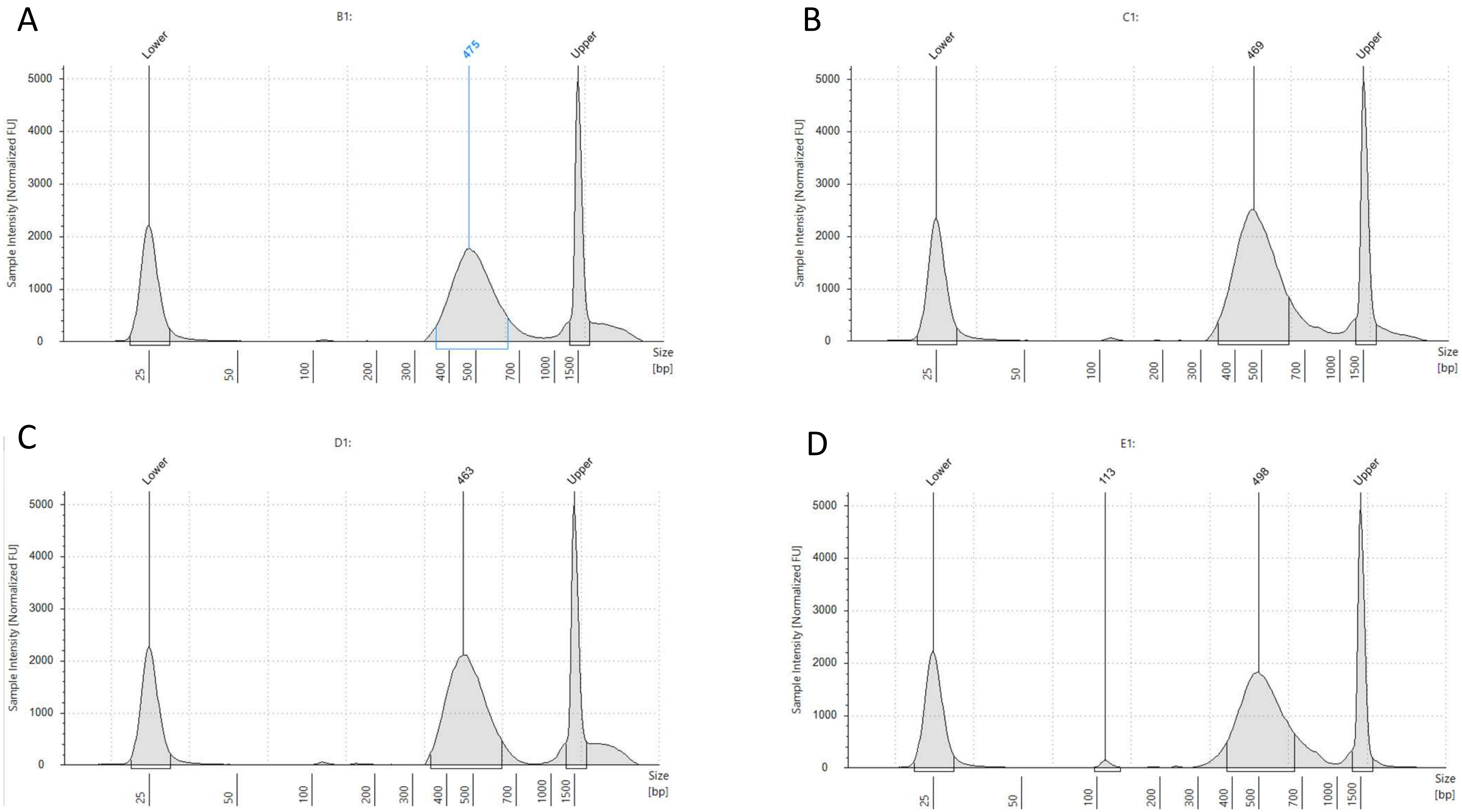

Suppl Figure 1

Figure S1. Quality control of prepared scRNA-seq libraries.
We present here electropherograms of CD34+lin-CD45- (A), CD133+lin-CD45- (B), CD34+lin-CD45+ (C) and CD133+lin-CD45+ (D) analyzed with the Agilent D5000 ScreenTape assay on the TapeStation 4150.
