# Supplementary material for: scRNA-seq revealed transcriptional signatures of human umbilical cord primitive stem cells and their germ lineage origin regulated by imprinted genes
Source: Sci Rep. 2024 Nov 26;14:29264. doi: 10.1038/s41598-024-79810-4 (PMC11589151; doi:10.1038/s41598-024-79810-4)
Supplement: Supplementary file 2 — Supplementary Information 2. [file 41598_2024_79810_MOESM2_ESM.pdf]

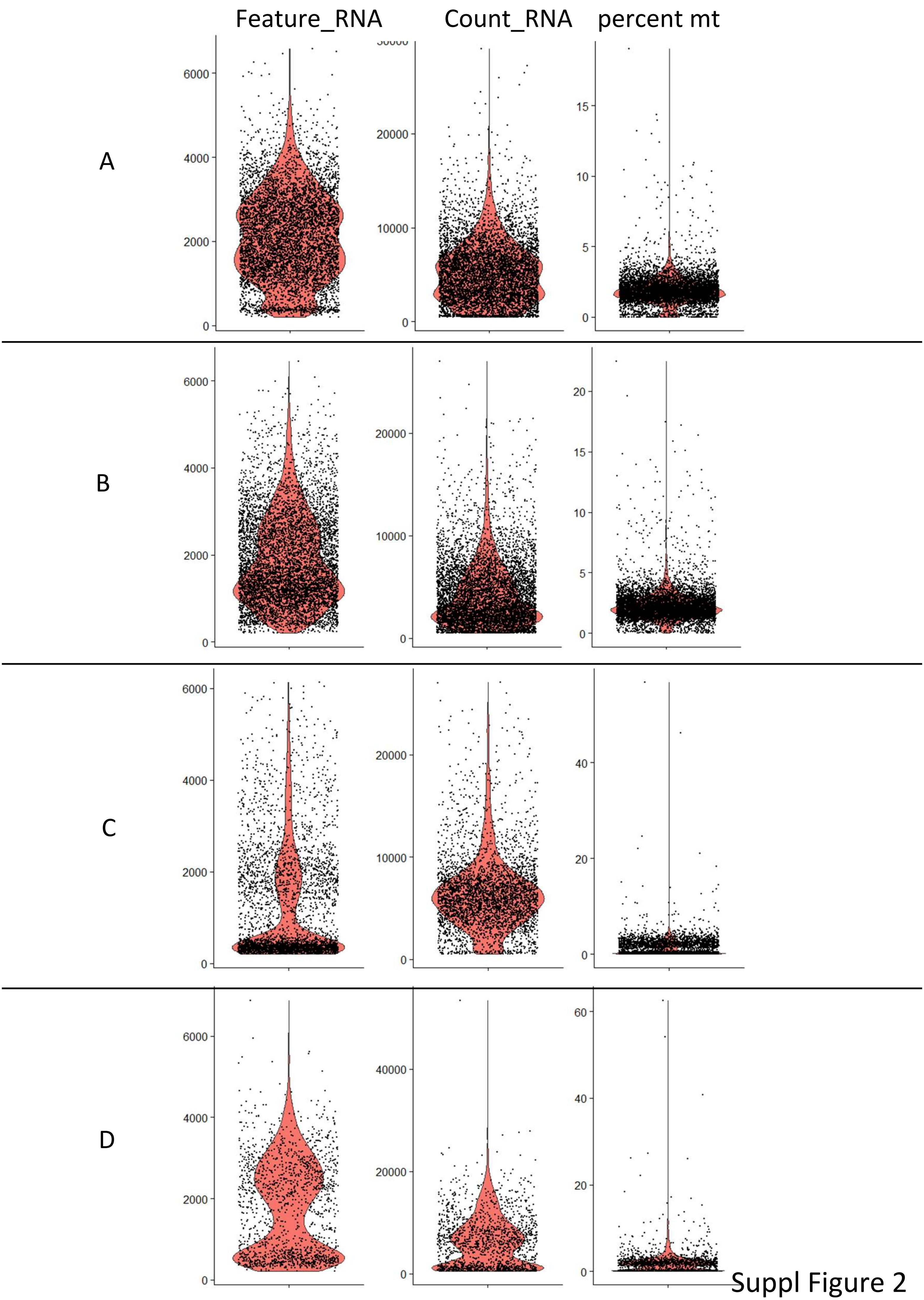

Suppl Figure 2

Figure S2. QC metrics presented as violin plots of the number of unique genes found in each cell (left panel), the number of RNA molecules detected within a cell (middle panel) and the percentage of reads which map to mitochondrial genome (right panel) of CD133+lin-CD45- (A), CD34+lin-CD45+ (B), CD133+lin-CD45- (C) and CD34+lin-CD45- (D).
