# Supplementary material for: scRNA-seq revealed transcriptional signatures of human umbilical cord primitive stem cells and their germ lineage origin regulated by imprinted genes
Source: Sci Rep. 2024 Nov 26;14:29264. doi: 10.1038/s41598-024-79810-4 (PMC11589151; doi:10.1038/s41598-024-79810-4)
Supplement: Supplementary file 3 — Supplementary Information 3. [file 41598_2024_79810_MOESM3_ESM.pdf]

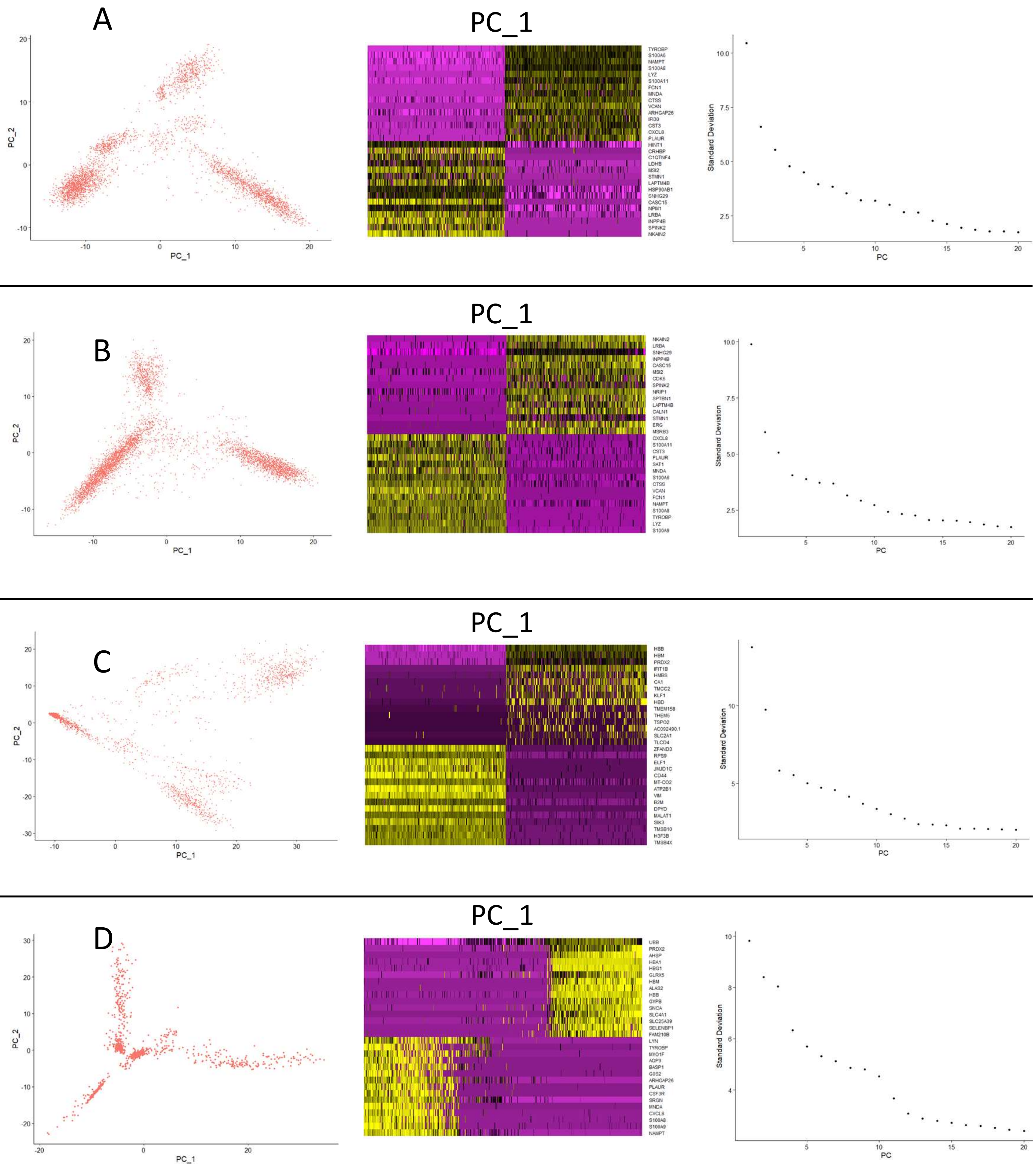

Figure S3. Dimensional reduction analysis of the dataset.
Dimension plot (left panel) and heatmap (middle panel) of first principal components based on the list of most positive and negative markers as well as an elbow plot presenting the ranking of principle components based on the percentage of variance explained by each one (right panel) of CD133+lin-CD45- (A), CD34+lin-CD45+ (B), CD133+lin-CD45- (C) and CD34+lin-CD45- (D).
