# Supplementary material for: scRNA-seq revealed transcriptional signatures of human umbilical cord primitive stem cells and their germ lineage origin regulated by imprinted genes
Source: Sci Rep. 2024 Nov 26;14:29264. doi: 10.1038/s41598-024-79810-4 (PMC11589151; doi:10.1038/s41598-024-79810-4)
Supplement: Supplementary file 4 — Supplementary Information 4. [file 41598_2024_79810_MOESM4_ESM.pdf]

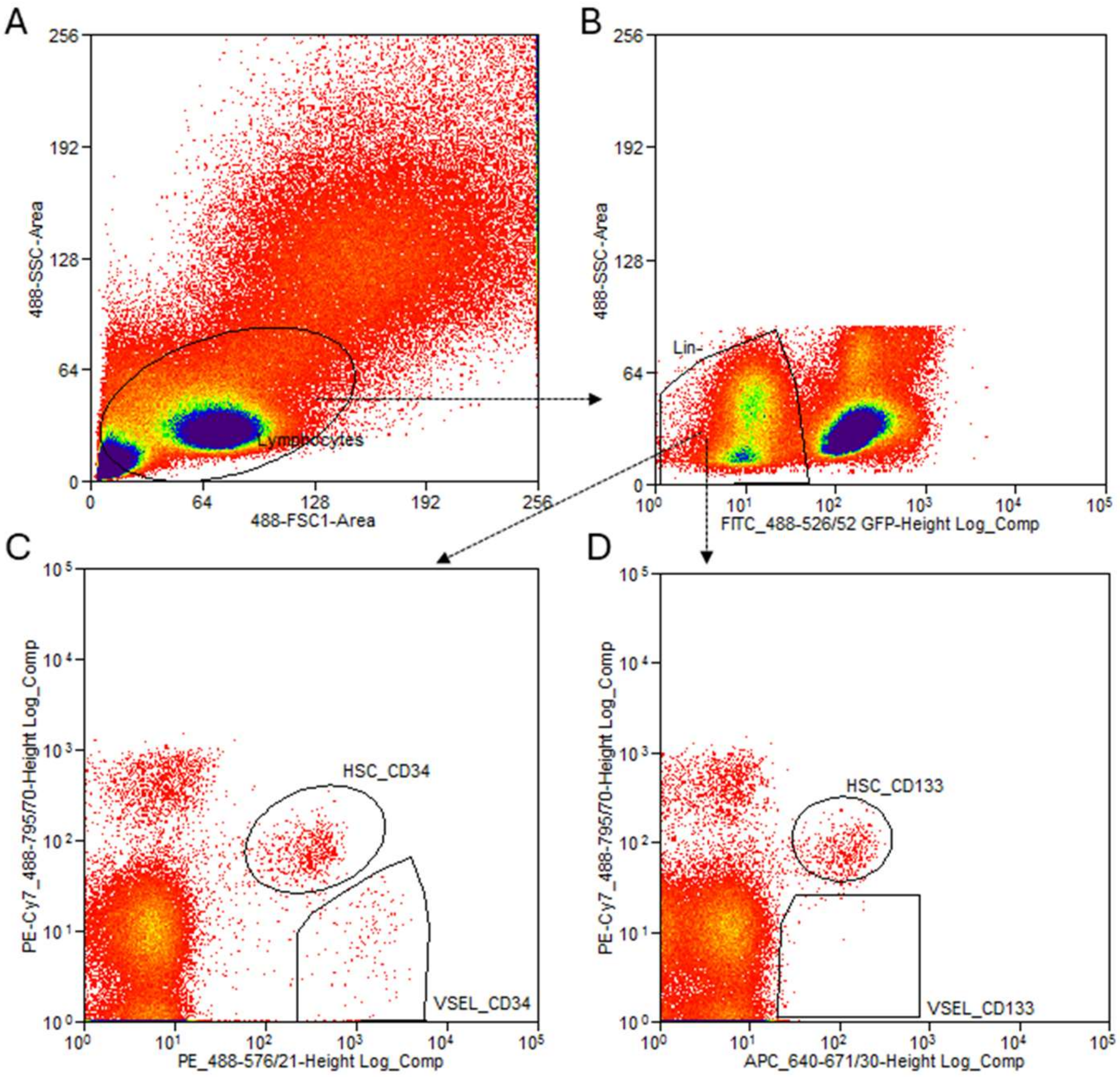

Suppl Figure 4

Figure S4. Gating strategy used for the isolation of VSELs and HSCs by Flourescence Activated Cell Sorting. Immunostained cells were first visualized by dot plot showing forward scatter (FSC) vs. side scatter (SSC) signals, where small events ranging from 2–15 µm were gated (Lymphocytes) (A) and further analyzed for the expression of Lineage markers. Lineage negative events were gated (Lin-) (B) and separately analyzed for the expression of CD34 and CD45 (C) as well as CD133 and CD45 (D) antigens. The populations of CD34+Lin-CD45- VSELs (VSEL_CD34), CD34+Lin-CD45+ HSCs (HSC_CD34) (C) and CD133+Lin-CD45- VSELs (VSEL_CD133) and CD133+Lin-CD45+ HSCs (HSC_CD133) (D) were sorted. MNCs isolation and staining was described in Materials and Methods section. Representative dot plots saved during the sample acquisition are shown.
