# Supplementary material for: scRNA-seq revealed transcriptional signatures of human umbilical cord primitive stem cells and their germ lineage origin regulated by imprinted genes
Source: Sci Rep. 2024 Nov 26;14:29264. doi: 10.1038/s41598-024-79810-4 (PMC11589151; doi:10.1038/s41598-024-79810-4)
Supplement: Supplementary file 5 — Supplementary Information 5. [file 41598_2024_79810_MOESM6_ESM.pdf]

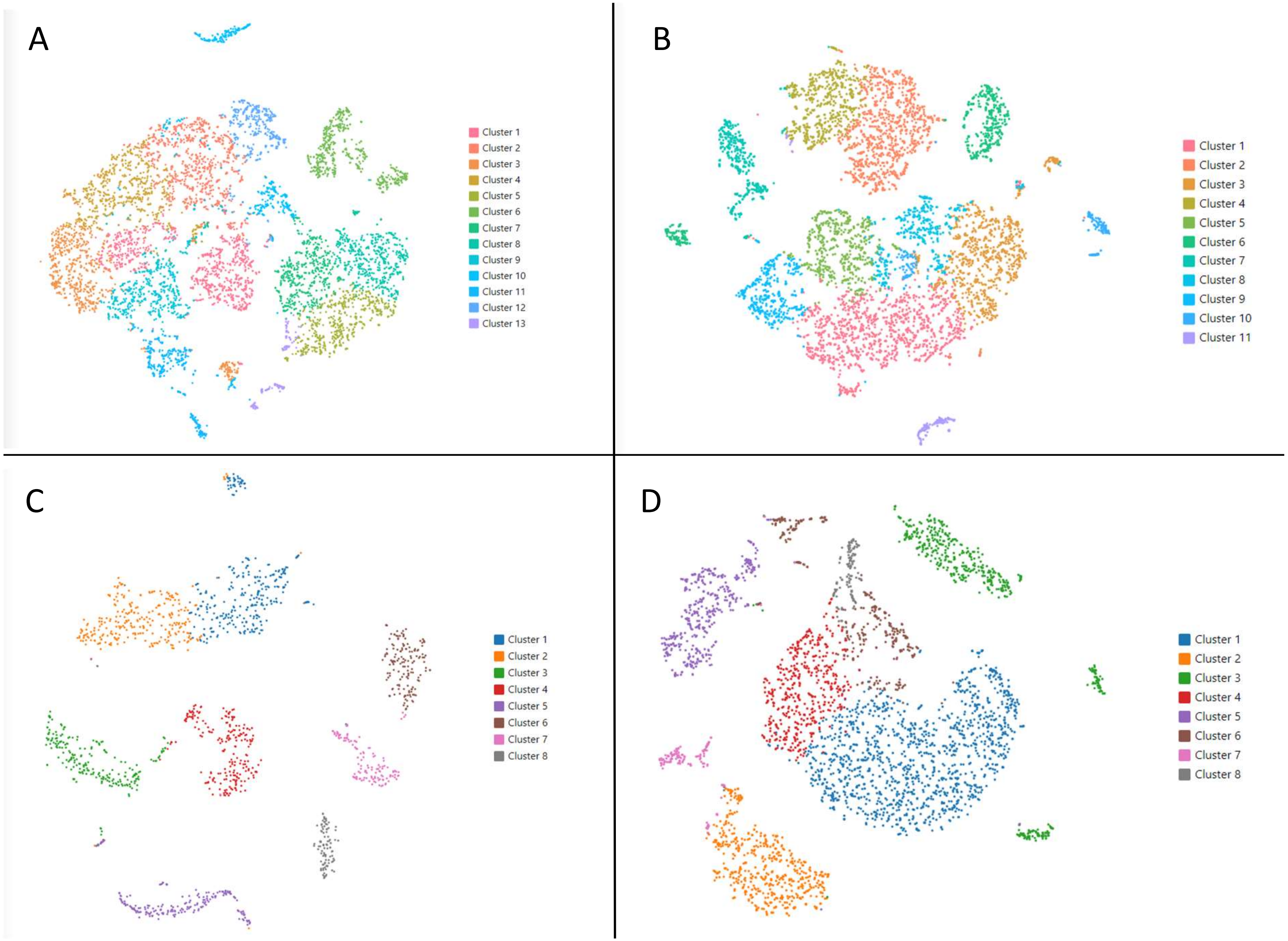

Suppl Figure 5

Figure S5. Identification of subpopulations in all studied samples of HSCs (A and B) - CD34+Lin-CD45+ (A), CD133+Lin-CD45+ (B), and VSELs (C and D) CD34+Lin-CD45- (C) and CD133+Lin-CD45- (D) visualized by UMAP method. We identified several cell subpopulations, namely 13 in CD34+Lin-CD45+ (A), 11 in CD133+Lin-CD45+ (B) and 8 in both CD34+Lin-CD45- (C) and CD133+Lin-CD45- (D).
